# Supplementary material for: Evidence that G-quadruplexes form in pathogenic fungi and represent promising antifungal targets
Source: EMBO Mol Med. 2025 Nov 17;17(12):3636–56. doi: 10.1038/s44321-025-00340-1 (PMC12686049; doi:10.1038/s44321-025-00340-1)
Supplement: Supplementary file 1 — Appendix [file 44321_2025_340_MOESM1_ESM.pdf]

**Appendix**

**Table of Content**

Appendix Table S1……………………………………………………………………….2

Appendix Table S2……………………………………………………………………….2

Appendix Figure S1………………………………………………………………………3

Appendix Figure S2………………………………………………………………………4

Appendix Figure S3………………………………………………………………………5

Appendix Figure S4………………………………………………………………………6

Appendix Figure S5………………………………………………………………………7

Appendix Figure S6………………………………………………………………………8

Appendix Figure S7………………………………………………………………………9

Appendix Figure S8……………………………………………………………………..10

Appendix Figure S9……………………………………………………………………..10

Appendix Figure S10……………………………………………………………………11

References………………………………………………………………………………12

**Appendix Table S1.** The fungal isolates used in this study and minimum inhibitory concentrations

| | | $MIC_{50/90}$ (μM) | $MIC_{50/90}$ (μM) | |
|---|---|---|---|---|
| **Species** | **Strain** | **PhenDC3** | **PDS** | **Source** |
| *A. fumigatus* | 22M7007854 | 0.83/1.56 | 6.96/12.50 | This study |
| *A. fumigatus* | 22M7004177 | 0.40/0.78 | 7.93/12.50 | This study |
| *A. fumigatus* | CEA10 | 0.89/3.13 | 5.38/6.25 | [1] |
| *A. fumigatus* | A1160+ | 1.13/3.13 | 3.31/6.25 | [2] |
| *A. fumigatus* | $TR_{34}$/L98H | 0.38/0.78 | 5.95/12.50 | [3] |
| *A. hiratsukae* | 22M7007855 | 1.10/>12.50 | 3.98/6.25 | This study |
| *A. udagawae* | 22M7007856 | 0.69/6.25 | 6.09/12.50 | This study |
| *A. flavus* | 22M8001325 | ND/ND | ND/ND | This study |
| *A. brasiliensis* | ATCC16404 | ND/ND | 11.43/12.50 | [4] |
| *A. niger* | 22M8001245 | ND/ND | 9.12/12.50 | This study |
| *C. albicans* | JC747 | ND/ND | 5.94/12.5 | [5] |
| *C. auris* | NCPF8985 | 30.27/50 | 5.48/6.25 | [6] |
| *C. glabrata* | CBS-138 | ND/ND | 3.94/6.25 | [7] |

ND = Not determined

**Appendix Table S2.** The number of predicted G4s in the *A. fumigatus* genome and whether these are in the gene bodies or intergenic regions

| Chromosome | Total bp | Total G4s | G4s/kbp | G4s in gene | % G4s | Intergenic G4s | % G4s |
|---|---|---|---|---|---|---|---|
| 1 | 4918979 | 7669 | 1.56 | 4898 | 63.87 | 2749 | 35.85 |
| 2 | 4844472 | 7870 | 1.62 | 5250 | 66.71 | 2599 | 33.02 |
| 3 | 4079167 | 6539 | 1.60 | 4257 | 65.10 | 2263 | 34.61 |
| 4 | 3923705 | 6015 | 1.53 | 3865 | 64.26 | 2138 | 35.54 |
| 5 | 3948441 | 6087 | 1.54 | 4069 | 66.85 | 1993 | 32.74 |
| 6 | 3778736 | 5811 | 1.54 | 3742 | 64.40 | 2041 | 35.12 |
| 7 | 2058334 | 3053 | 1.48 | 1848 | 60.53 | 1184 | 38.78 |
| 8 | 1833124 | 2627 | 1.43 | 1625 | 61.86 | 994 | 37.84 |

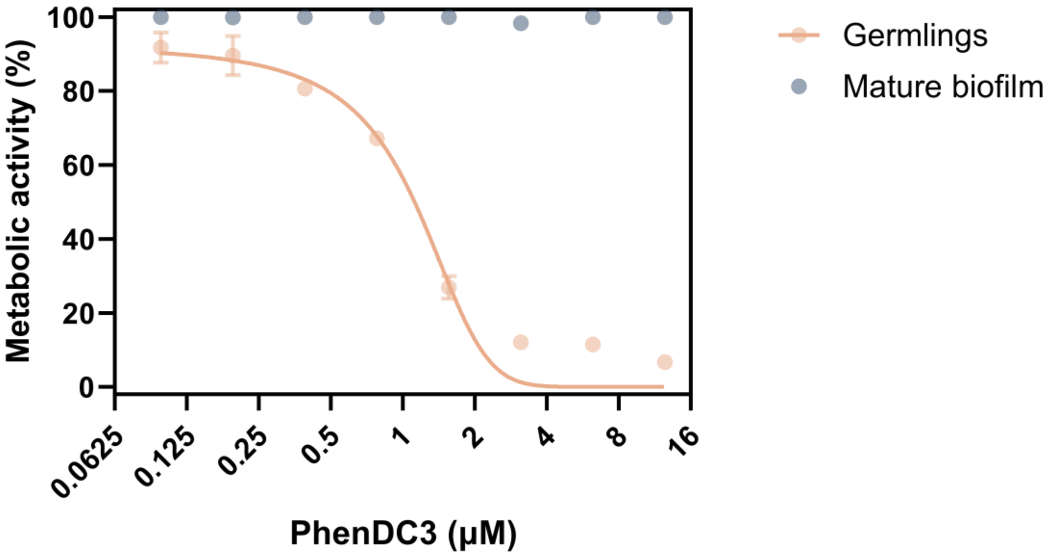


**Appendix Fig. S1.** *A. fumigatus* germlings (6 h of growth) or mature biofilms (<24 h growth) were treated with the indicated concentrations of PhenDC3 for 48 h at 37 °C. Metabolic activity was calculated as percentage of the untreated control. Experiments are in biological triplicate with each data point consisting of the mean of three technical replicates ± SEM.

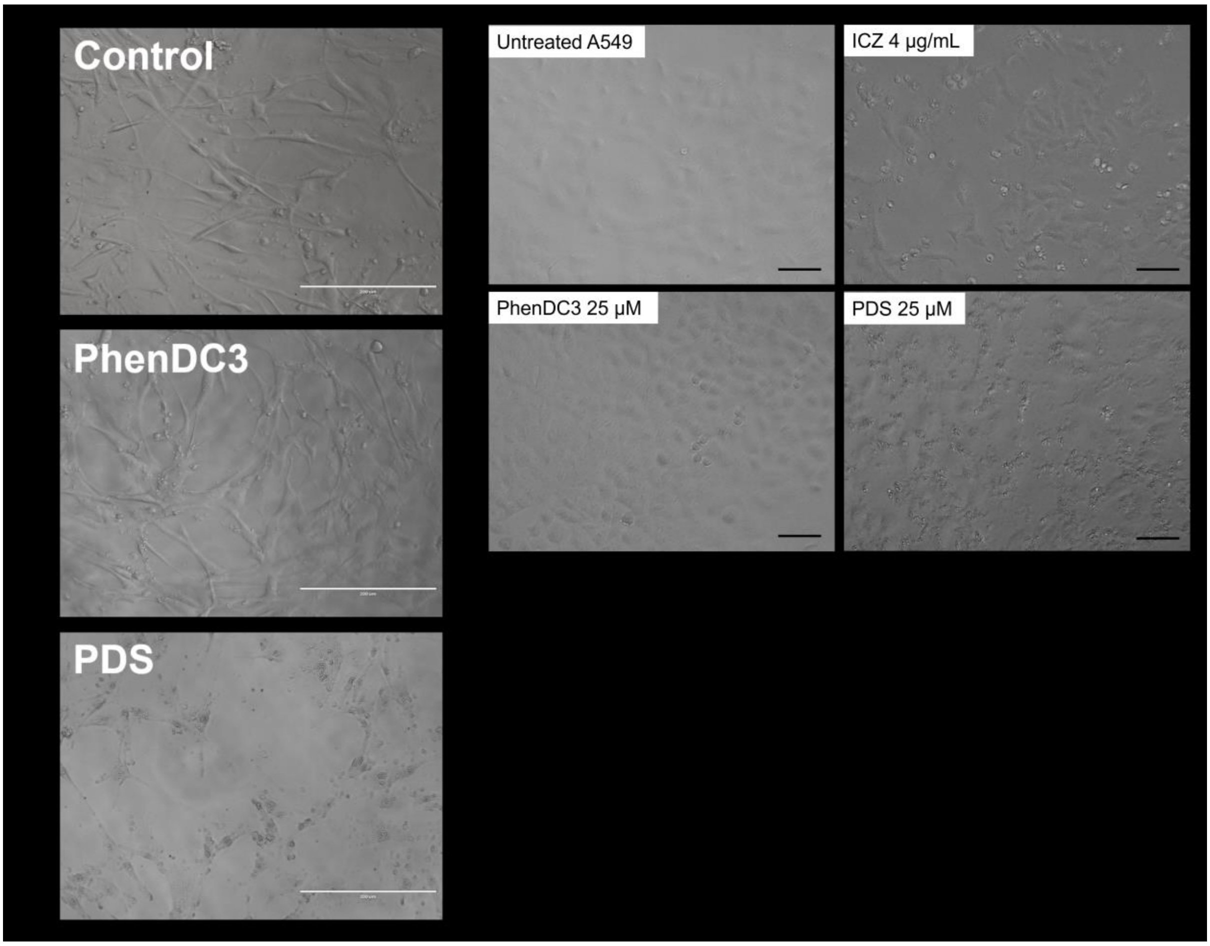


**Appendix Fig. S2.** Representative light microscopy images of (**a**) primary human vascular smooth muscle cells treated with 25 µM PhenDC3 or PDS for 24 h. Scale bars represent 200 µm. Representative light microscopy images of (**b**) human A549 lung cells treated with 25 µM PhenDC3, 25 µM PDS, or 4 µg/mL itraconazole (ICZ) for 24 h. Scale bars represent 100 µm.

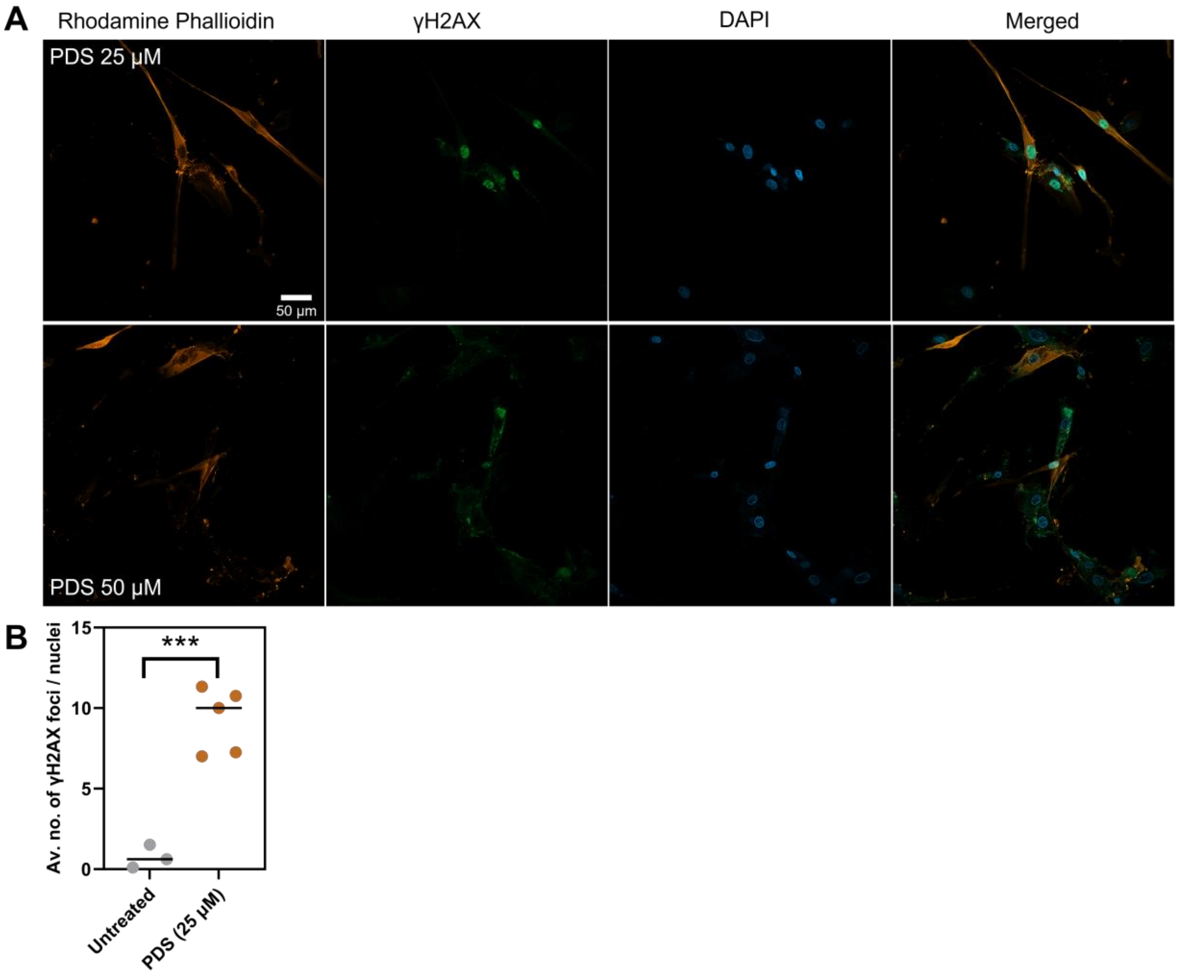


**Appendix Fig. S3. (A)** Representative confocal microscopy images of primary human vascular smooth muscle cells treated with 25 µM or 50 µM PDS for 24 h. Scale bars represent 50 µm. Cells were incubated with rhodamine phalloidin, a γH2AX antibody, or DAPI to visualise F-actin, DNA damage, or the nucleus, respectively. Images were obtained using a Zeiss LSM 980 laser scanning confocal microscope equipped with an Airyscan 2 detector using the 20x objective. Images were analysed using Fiji 1.53t. (**B**) Quantification of the γH2AX-foci following treatment with 25 µM PDS.

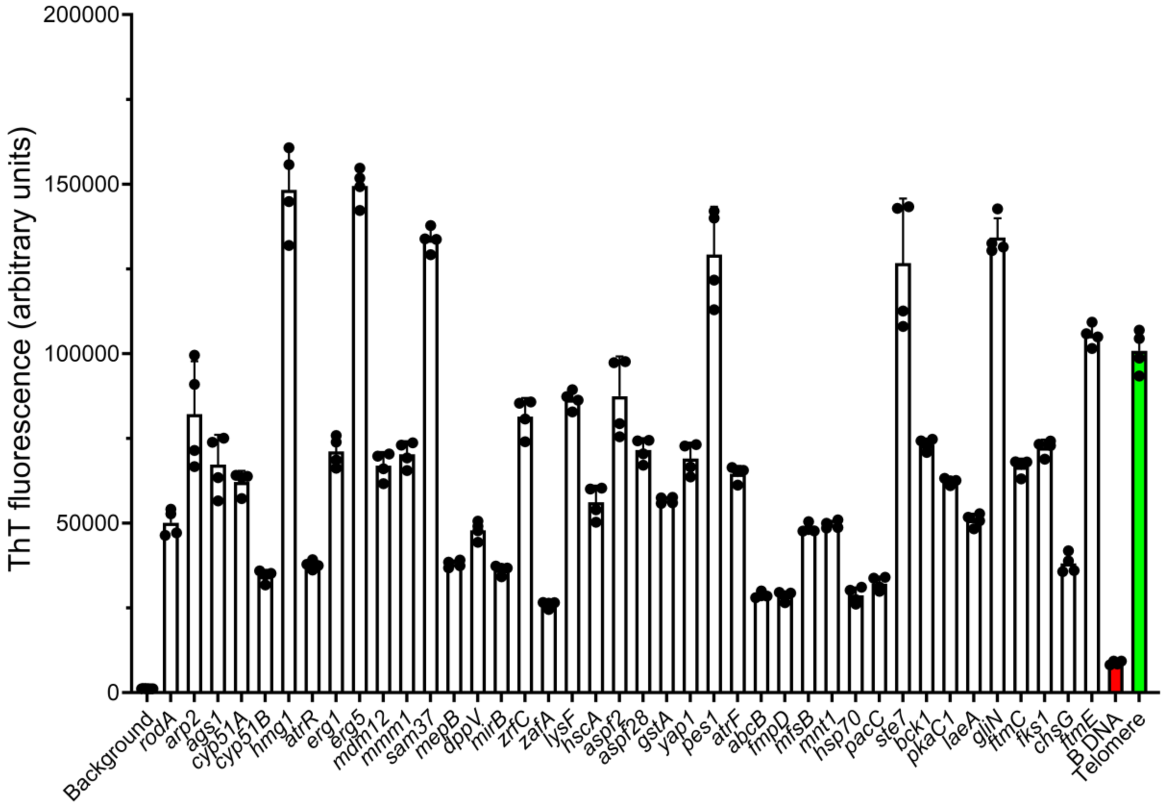


**Appendix Fig. S4.** Thioflavin T fluorescence following the binding to G4s that have formed in single-stranded DNA sequences which have been annealed in a buffer containing 10 mM sodium cacodylate and 100 mM KCl. Sequences are representative of those identified in a range of genes involved in growth, virulence, and drug resistance. B-DNA unable to form a G4 was used as a negative control and the $[GGGTTA]_4$ telomeric repeat sequence was used as a positive control.

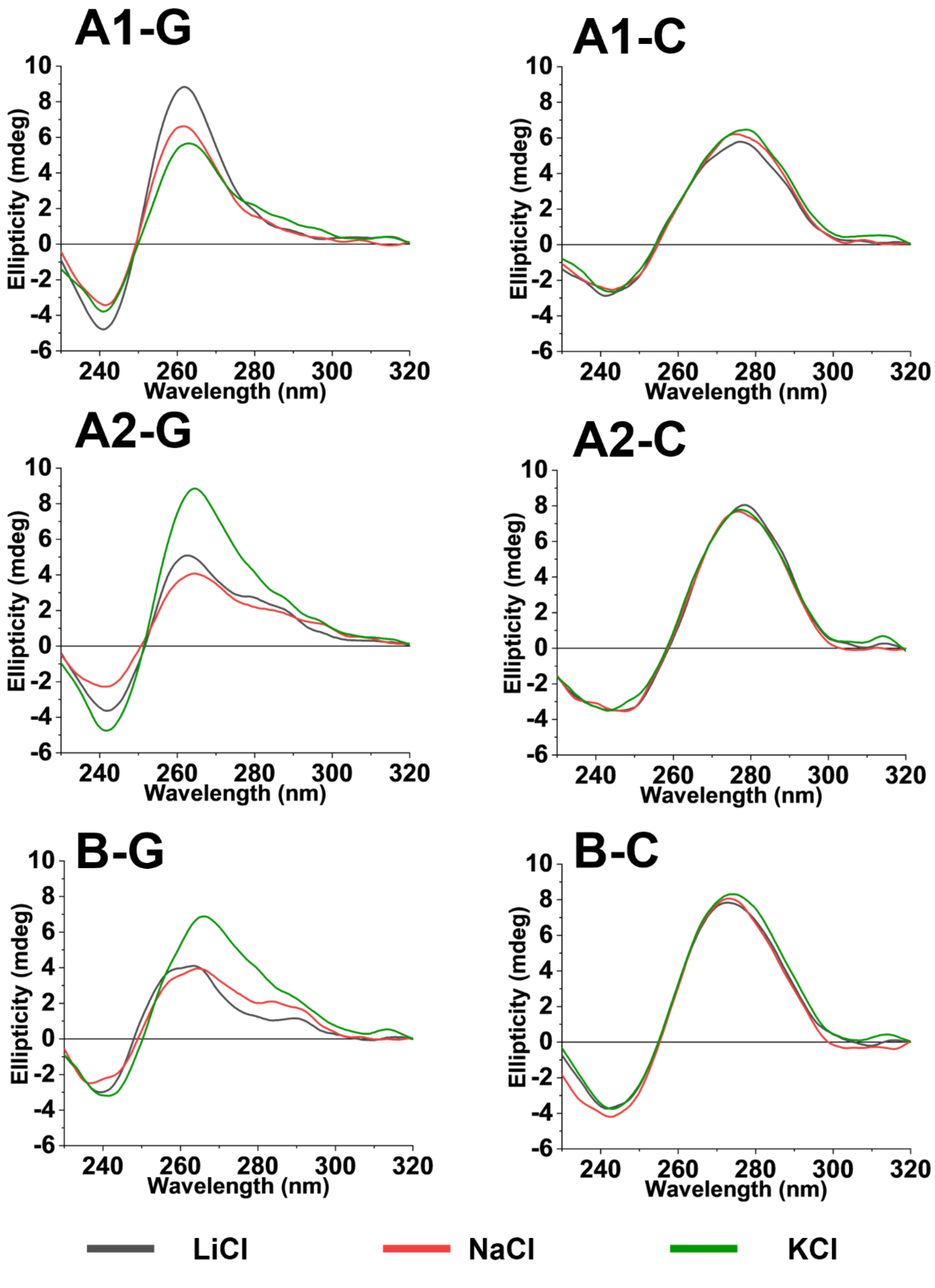


**Appendix Fig. S5.** CD Spectroscopy of the *cyp51* sequences A1-G, A1-C, A2-G, A2-C, B-G, and B-C. DNA oligonucleotides (10 µM) were annealed in 10 mM sodium cacodylate buffer containing 100 mM LiCl, NaCl, or KCl (as indicated) at pH 7.0.

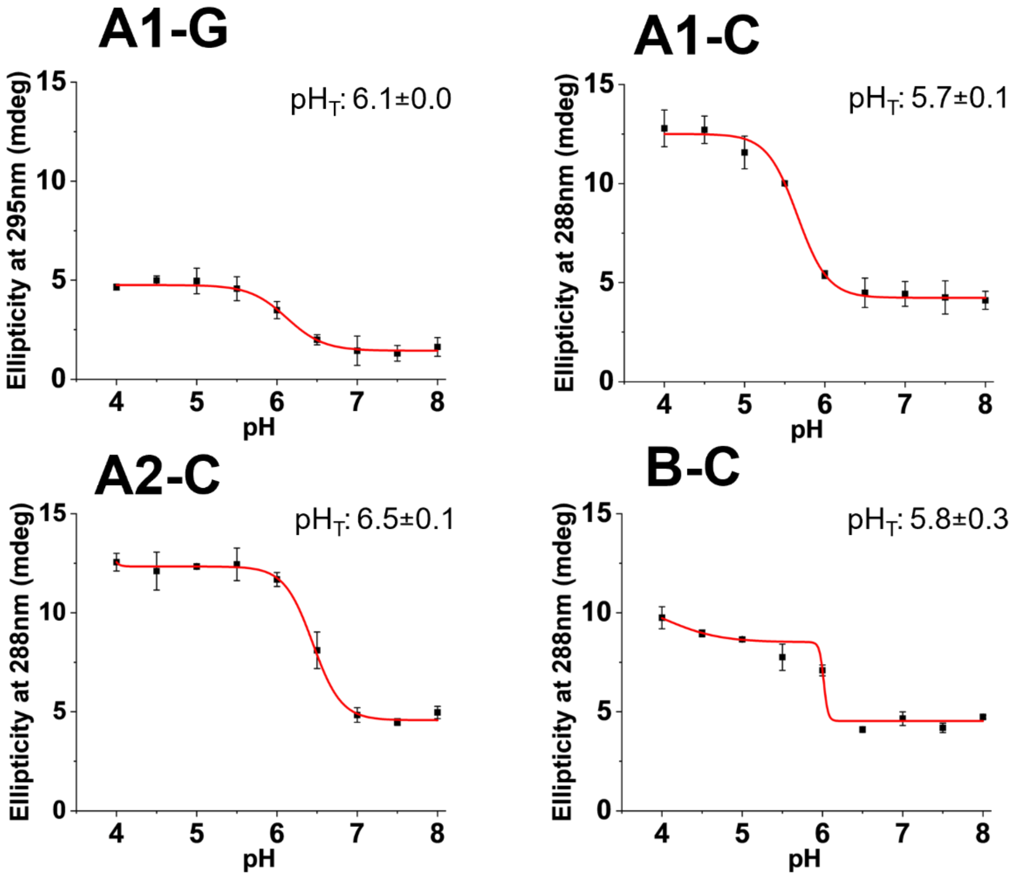


**Appendix Fig. S6.** Corresponding plot ellipticity for the CD experiments in Fig. 1c. at 288nm at the different pH. This plot was used to determine the transitional pH ($pH_T$) from the inflection point of a Boltzmann sigmoidal.

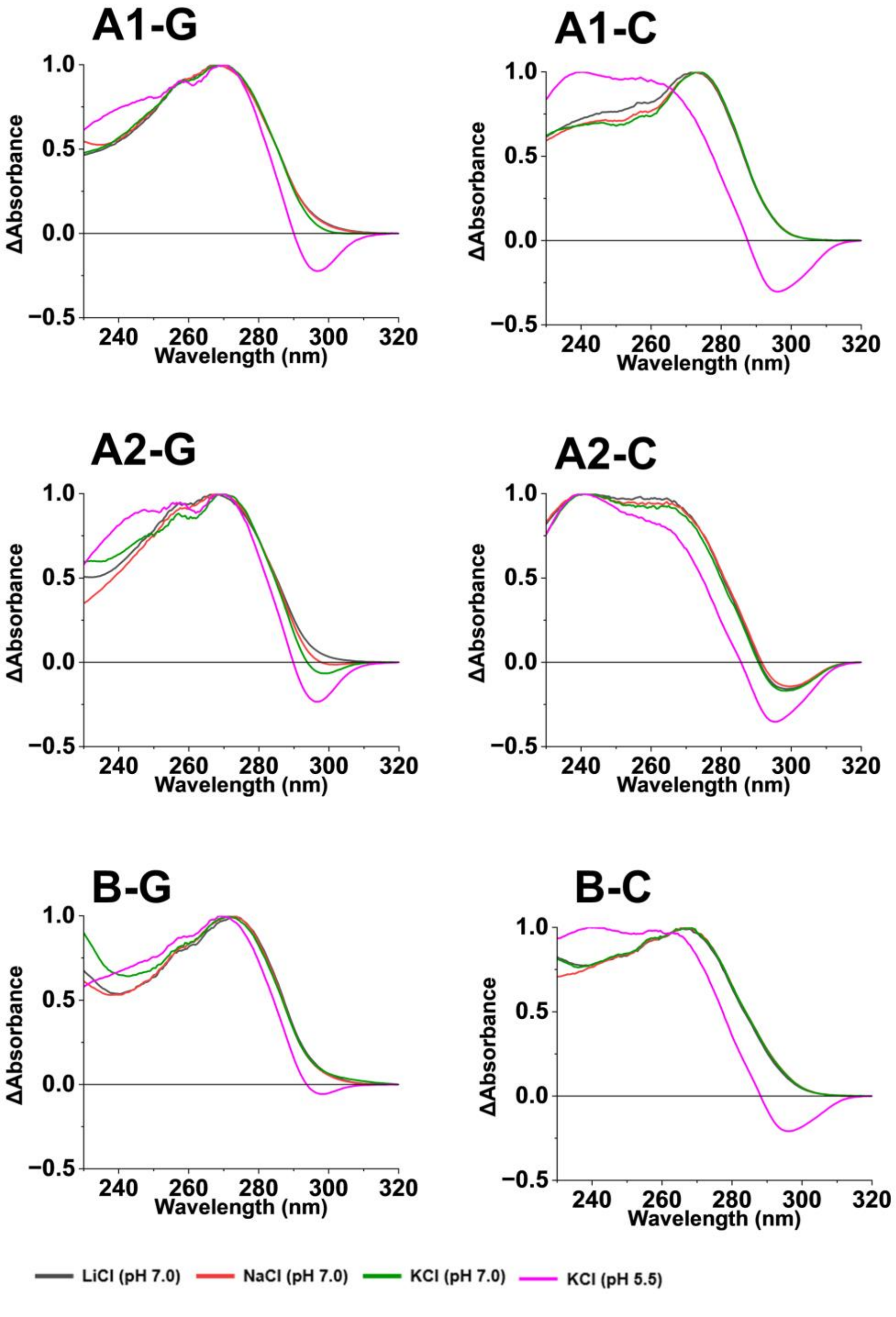


**Appendix Fig. S7.** Thermal difference spectra of the *cyp51* sequences. DNA oligonucleotides (5 µM) were annealed in 10 mM sodium cacodylate buffer containing 100 mM LiCl, NaCl, KCl (pH 7.0), or KCl (pH 5.5) as indicated.

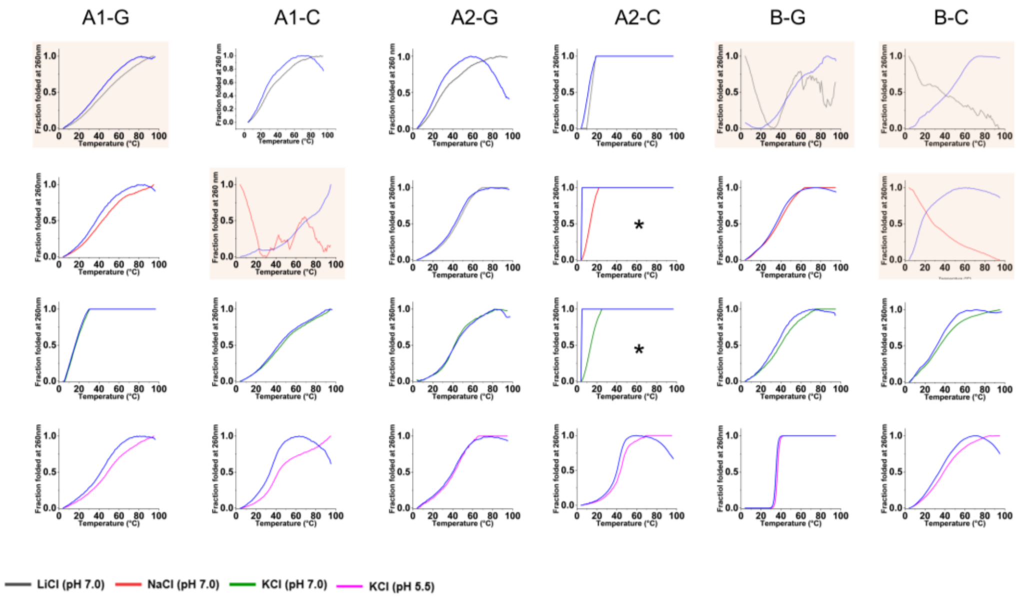


**Appendix Fig S8**. Fraction folded UV melt and annealing profile of 5 µM of the DNA oligonucleotides A1-C, A1-G, A2-C, A2-G, B-C, and B-G at 260 nm in 10 mM sodium cacodylate buffer containing 100 mM LiCl, NaCl, KCl (all at pH 7.0) and KCl (at pH 5.5). Graphs highlighted in orange showed no repeatable thermodynamic profile at 260nm. Sequences demonstrating minimal annealing transitions (<4°C) are marked with an asterisk (*)

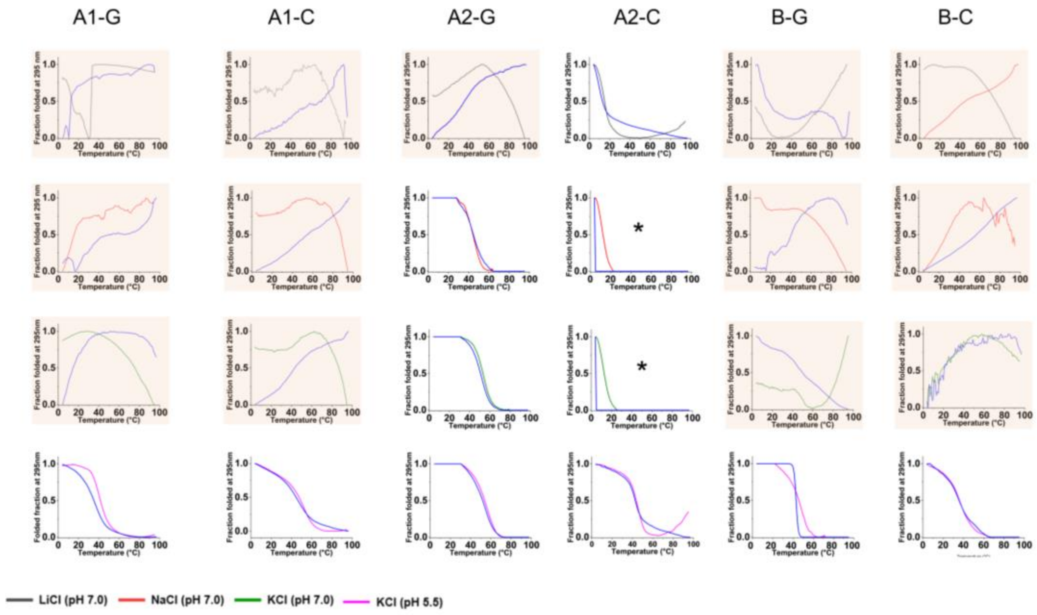


**Appendix Fig S9**. Fraction folded UV melt and annealing profile of 5 µM of the DNA oligonucleotides A1-C, A1-G, A2-C, A2-G, B-C, and B-G at 295 nm in 10 mM sodium cacodylate buffer containing 100 mM LiCl, NaCl, KCl (all at pH 7.0) and KCl (at pH 5.5). Graphs highlighted in orange showed no repeatable thermodynamic profile at 295nm. Sequences demonstrating minimal annealing transitions (<4°C) are marked with an asterisk (*).

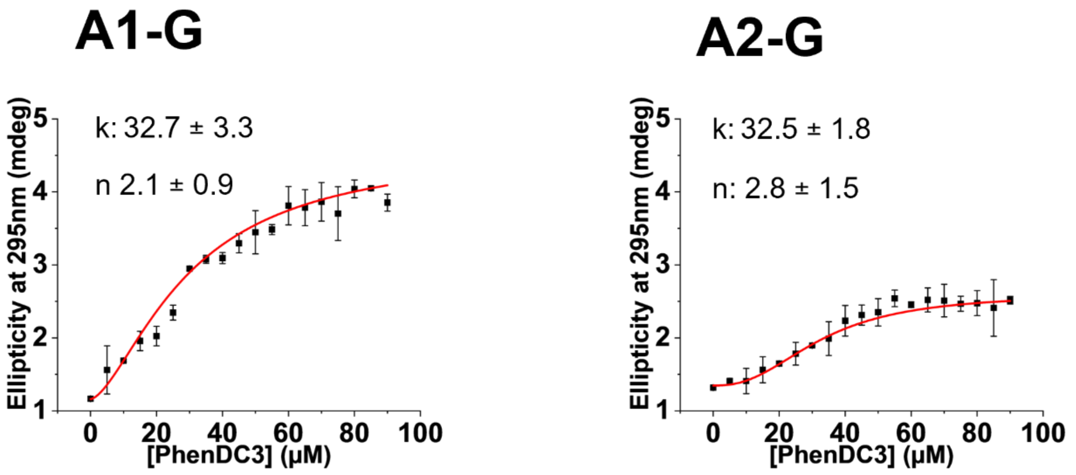


**Appendix Fig. S10.** Plot of ellipticity of the experimental repeats in Fig. 1d at 295nm against PhenDC3 concentration and the corresponding Hill-1 fitting.

**References**


1. Girardin H, Latgé JP, Srikantha T, Morrow B, Soll DR. Development of DNA probes for fingerprinting Aspergillus fumigatus. *J Clin Microbiol*. 1993;31(6):1547-1554. doi:10.1128/jcm.31.6.1547-1554.1993
2. Fraczek MG, Bromley M, Buied A, et al. The cdr1B efflux transporter is associated with non-cyp51a-mediated itraconazole resistance in Aspergillus fumigatus. *J Antimicrob Chemother*. 2013;68(7):1486-1496. doi:10.1093/jac/dkt075
3. Bromley MJ, van Muijlwijk G, Fraczek MG, et al. Occurrence of azole-resistant species of Aspergillus in the UK environment. *J Glob Antimicrob Resist*. 2014;2(4):276-279. doi:10.1016/j.jgar.2014.05.004
4. Varga J, Kocsubé S, Tóth B, et al. Aspergillus brasiliensis sp. nov., a biseriate black Aspergillus species with world-wide distribution. *Int J Syst Evol Microbiol*. 2007;57(Pt 8):1925-1932. doi:10.1099/ijs.0.65021-0
5. da Silva Dantas A, Patterson MJ, Smith DA, et al. Thioredoxin regulates multiple hydrogen peroxide-induced signaling pathways in Candida albicans. *Mol Cell Biol*. 2010;30(19):4550-4563. doi:10.1128/MCB.00313-10
6. Borman AM, Szekely A, Johnson EM. Isolates of the emerging pathogen Candida auris present in the UK have several geographic origins. *Med Mycol*. 2017;55(5):563-567. doi:10.1093/mmy/myw147
7. Dujon, B., Sherman, D., Fischer, G. *et al.* Genome evolution in yeasts. *Nature* **430**, 35–44 (2004). https://doi.org/10.1038/nature02579
