# Supplementary figures and images for: Evidence that G-quadruplexes form in pathogenic fungi and represent promising antifungal targets (part 1 of 2)
Source: EMBO Mol Med. 2025 Nov 17;17(12):3636–56. doi: 10.1038/s44321-025-00340-1 (PMC12686049; doi:10.1038/s44321-025-00340-1)

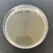

Supplement: Supplementary file 4 — Source data Fig. 2 [file 44321_2025_340_MOESM4_ESM.zip › Figure 2/Figure 2B/0.39 PHENDC3 24H.tif]

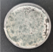

Supplement: Supplementary file 4 — Source data Fig. 2 [file 44321_2025_340_MOESM4_ESM.zip › Figure 2/Figure 2B/0.39 PHENDC3 48H.tif]

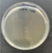

Supplement: Supplementary file 4 — Source data Fig. 2 [file 44321_2025_340_MOESM4_ESM.zip › Figure 2/Figure 2B/0.78 PHENDC3 24H.tif]

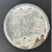

Supplement: Supplementary file 4 — Source data Fig. 2 [file 44321_2025_340_MOESM4_ESM.zip › Figure 2/Figure 2B/0.78 PHENDC3 48H.tif]

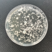

Supplement: Supplementary file 4 — Source data Fig. 2 [file 44321_2025_340_MOESM4_ESM.zip › Figure 2/Figure 2B/1.56 PDS 24H.tif]

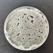

Supplement: Supplementary file 4 — Source data Fig. 2 [file 44321_2025_340_MOESM4_ESM.zip › Figure 2/Figure 2B/1.56 PDS 48H.tif]

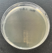

Supplement: Supplementary file 4 — Source data Fig. 2 [file 44321_2025_340_MOESM4_ESM.zip › Figure 2/Figure 2B/1.56 PHENDC3 24H.tif]

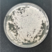

Supplement: Supplementary file 4 — Source data Fig. 2 [file 44321_2025_340_MOESM4_ESM.zip › Figure 2/Figure 2B/1.56 PHENDC3 48H.tif]

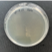

Supplement: Supplementary file 4 — Source data Fig. 2 [file 44321_2025_340_MOESM4_ESM.zip › Figure 2/Figure 2B/12.5 PDS 24H.tif]

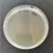

Supplement: Supplementary file 4 — Source data Fig. 2 [file 44321_2025_340_MOESM4_ESM.zip › Figure 2/Figure 2B/12.5 PDS 48H.tif]

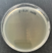

Supplement: Supplementary file 4 — Source data Fig. 2 [file 44321_2025_340_MOESM4_ESM.zip › Figure 2/Figure 2B/12.5 PHENDC3 24H.tif]

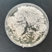

Supplement: Supplementary file 4 — Source data Fig. 2 [file 44321_2025_340_MOESM4_ESM.zip › Figure 2/Figure 2B/12.5 PHENDC3 48H.tif]

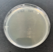

Supplement: Supplementary file 4 — Source data Fig. 2 [file 44321_2025_340_MOESM4_ESM.zip › Figure 2/Figure 2B/25 PDS 24H.tif]

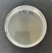

Supplement: Supplementary file 4 — Source data Fig. 2 [file 44321_2025_340_MOESM4_ESM.zip › Figure 2/Figure 2B/25 PDS 48H.tif]

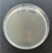

Supplement: Supplementary file 4 — Source data Fig. 2 [file 44321_2025_340_MOESM4_ESM.zip › Figure 2/Figure 2B/25 PHENDC3 24H.tif]

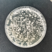

Supplement: Supplementary file 4 — Source data Fig. 2 [file 44321_2025_340_MOESM4_ESM.zip › Figure 2/Figure 2B/25 PHENDC3 48H.tif]

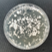

Supplement: Supplementary file 4 — Source data Fig. 2 [file 44321_2025_340_MOESM4_ESM.zip › Figure 2/Figure 2B/3.13 PDS 24H.tif]

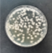

Supplement: Supplementary file 4 — Source data Fig. 2 [file 44321_2025_340_MOESM4_ESM.zip › Figure 2/Figure 2B/3.13 PDS 48H.tif]

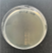

Supplement: Supplementary file 4 — Source data Fig. 2 [file 44321_2025_340_MOESM4_ESM.zip › Figure 2/Figure 2B/3.13 PHENDC3 24H.tif]

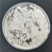

Supplement: Supplementary file 4 — Source data Fig. 2 [file 44321_2025_340_MOESM4_ESM.zip › Figure 2/Figure 2B/3.13 PHENDC3 48H.tif]

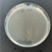

Supplement: Supplementary file 4 — Source data Fig. 2 [file 44321_2025_340_MOESM4_ESM.zip › Figure 2/Figure 2B/50 PDS 24H.tif]

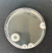

Supplement: Supplementary file 4 — Source data Fig. 2 [file 44321_2025_340_MOESM4_ESM.zip › Figure 2/Figure 2B/50 PDS 48H.tif]

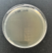

Supplement: Supplementary file 4 — Source data Fig. 2 [file 44321_2025_340_MOESM4_ESM.zip › Figure 2/Figure 2B/50 PHENDC3 24H.tif]

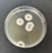

Supplement: Supplementary file 4 — Source data Fig. 2 [file 44321_2025_340_MOESM4_ESM.zip › Figure 2/Figure 2B/6.25 PDS 48H.tif]

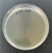

Supplement: Supplementary file 4 — Source data Fig. 2 [file 44321_2025_340_MOESM4_ESM.zip › Figure 2/Figure 2B/6.25 PHENDC3 24H.tif]

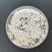

Supplement: Supplementary file 4 — Source data Fig. 2 [file 44321_2025_340_MOESM4_ESM.zip › Figure 2/Figure 2B/6.25 PHENDC3 48H.tif]

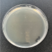

Supplement: Supplementary file 4 — Source data Fig. 2 [file 44321_2025_340_MOESM4_ESM.zip › Figure 2/Figure 2B/AMB 24H.tif]

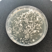

Supplement: Supplementary file 4 — Source data Fig. 2 [file 44321_2025_340_MOESM4_ESM.zip › Figure 2/Figure 2B/AMB 48H.tif]

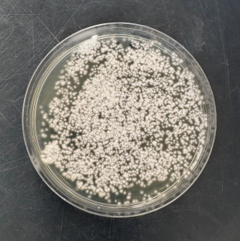

Supplement: Supplementary file 4 — Source data Fig. 2 [file 44321_2025_340_MOESM4_ESM.zip › Figure 2/Figure 2D/0.5 PDS 24H.tif]

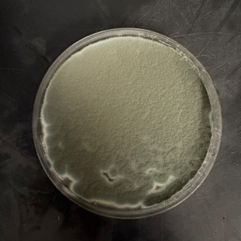

Supplement: Supplementary file 4 — Source data Fig. 2 [file 44321_2025_340_MOESM4_ESM.zip › Figure 2/Figure 2D/0.5 PDS 48H.tif]

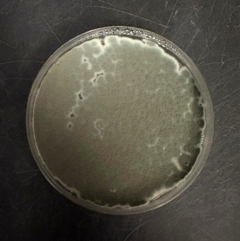

Supplement: Supplementary file 4 — Source data Fig. 2 [file 44321_2025_340_MOESM4_ESM.zip › Figure 2/Figure 2D/0.5 PHEN 48.tif]

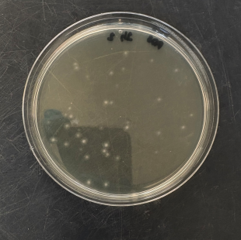

Supplement: Supplementary file 4 — Source data Fig. 2 [file 44321_2025_340_MOESM4_ESM.zip › Figure 2/Figure 2D/1 PDS 24H.tif]

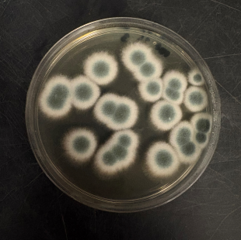

Supplement: Supplementary file 4 — Source data Fig. 2 [file 44321_2025_340_MOESM4_ESM.zip › Figure 2/Figure 2D/1 PDS 48H.tif]

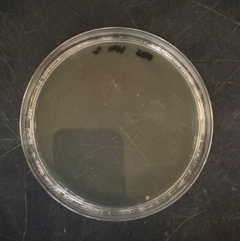

Supplement: Supplementary file 4 — Source data Fig. 2 [file 44321_2025_340_MOESM4_ESM.zip › Figure 2/Figure 2D/10 PDS 24H.tif]

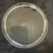

Supplement: Supplementary file 4 — Source data Fig. 2 [file 44321_2025_340_MOESM4_ESM.zip › Figure 2/Figure 2D/10 PHEN 24.tif]

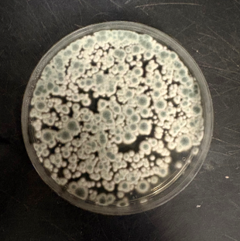

Supplement: Supplementary file 4 — Source data Fig. 2 [file 44321_2025_340_MOESM4_ESM.zip › Figure 2/Figure 2D/10 PHEN 48.tif]

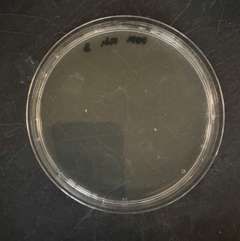

Supplement: Supplementary file 4 — Source data Fig. 2 [file 44321_2025_340_MOESM4_ESM.zip › Figure 2/Figure 2D/12 PDS 24H.tif]

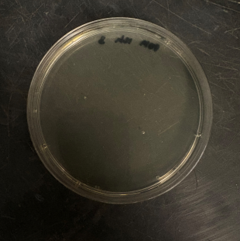

Supplement: Supplementary file 4 — Source data Fig. 2 [file 44321_2025_340_MOESM4_ESM.zip › Figure 2/Figure 2D/12 PDS 48H.tif]

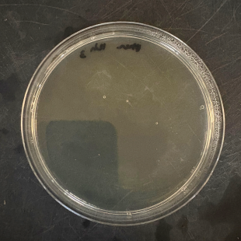

Supplement: Supplementary file 4 — Source data Fig. 2 [file 44321_2025_340_MOESM4_ESM.zip › Figure 2/Figure 2D/12 PHEN 24.tif]

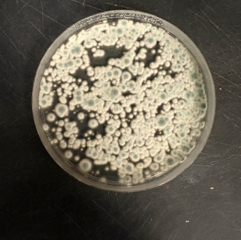

Supplement: Supplementary file 4 — Source data Fig. 2 [file 44321_2025_340_MOESM4_ESM.zip › Figure 2/Figure 2D/12 PHEN 48.tif]

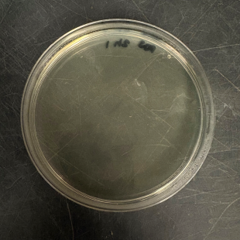

Supplement: Supplementary file 4 — Source data Fig. 2 [file 44321_2025_340_MOESM4_ESM.zip › Figure 2/Figure 2D/2.5 PDS 48H.tif]

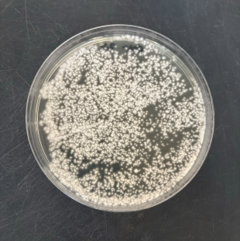

Supplement: Supplementary file 4 — Source data Fig. 2 [file 44321_2025_340_MOESM4_ESM.zip › Figure 2/Figure 2D/2.5 PHEN 24.tif]

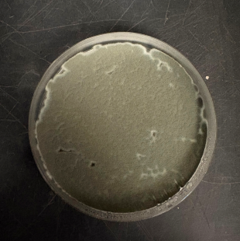

Supplement: Supplementary file 4 — Source data Fig. 2 [file 44321_2025_340_MOESM4_ESM.zip › Figure 2/Figure 2D/2.5 PHEN 48.tif]

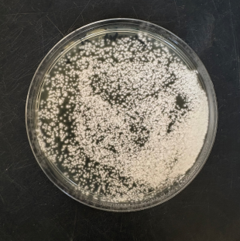

Supplement: Supplementary file 4 — Source data Fig. 2 [file 44321_2025_340_MOESM4_ESM.zip › Figure 2/Figure 2D/24H PRE.tif]

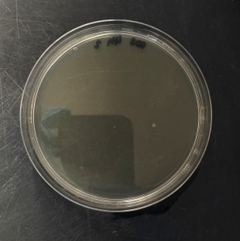

Supplement: Supplementary file 4 — Source data Fig. 2 [file 44321_2025_340_MOESM4_ESM.zip › Figure 2/Figure 2D/4 PDS 24H.tif]

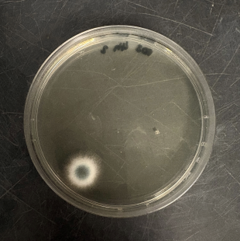

Supplement: Supplementary file 4 — Source data Fig. 2 [file 44321_2025_340_MOESM4_ESM.zip › Figure 2/Figure 2D/4 PDS 48H.tif]

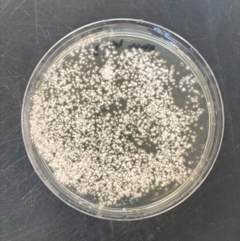

Supplement: Supplementary file 4 — Source data Fig. 2 [file 44321_2025_340_MOESM4_ESM.zip › Figure 2/Figure 2D/4 PHEN 24.tif]

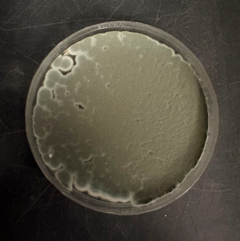

Supplement: Supplementary file 4 — Source data Fig. 2 [file 44321_2025_340_MOESM4_ESM.zip › Figure 2/Figure 2D/4 PHEN 48.tif]

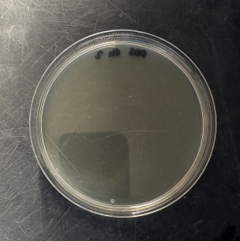

Supplement: Supplementary file 4 — Source data Fig. 2 [file 44321_2025_340_MOESM4_ESM.zip › Figure 2/Figure 2D/6 PDS 24H.tif]

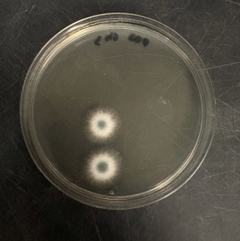

Supplement: Supplementary file 4 — Source data Fig. 2 [file 44321_2025_340_MOESM4_ESM.zip › Figure 2/Figure 2D/6 PDS 48H.tif]

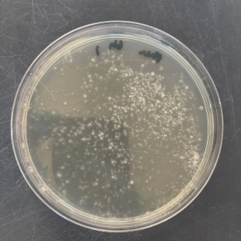

Supplement: Supplementary file 4 — Source data Fig. 2 [file 44321_2025_340_MOESM4_ESM.zip › Figure 2/Figure 2D/6 PHEN 24.tif]

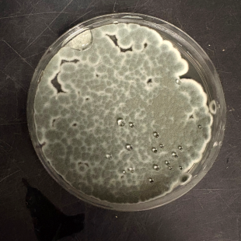

Supplement: Supplementary file 4 — Source data Fig. 2 [file 44321_2025_340_MOESM4_ESM.zip › Figure 2/Figure 2D/6 PHEN 48.tif]

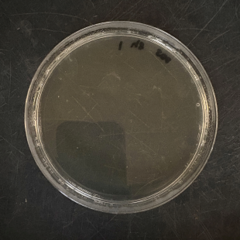

Supplement: Supplementary file 4 — Source data Fig. 2 [file 44321_2025_340_MOESM4_ESM.zip › Figure 2/Figure 2D/8 PDS 24H.tif]

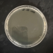

Supplement: Supplementary file 4 — Source data Fig. 2 [file 44321_2025_340_MOESM4_ESM.zip › Figure 2/Figure 2D/8 PHEN 24.tif]

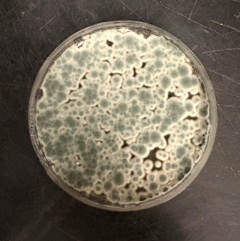

Supplement: Supplementary file 4 — Source data Fig. 2 [file 44321_2025_340_MOESM4_ESM.zip › Figure 2/Figure 2D/8 PHEN 48.tif]

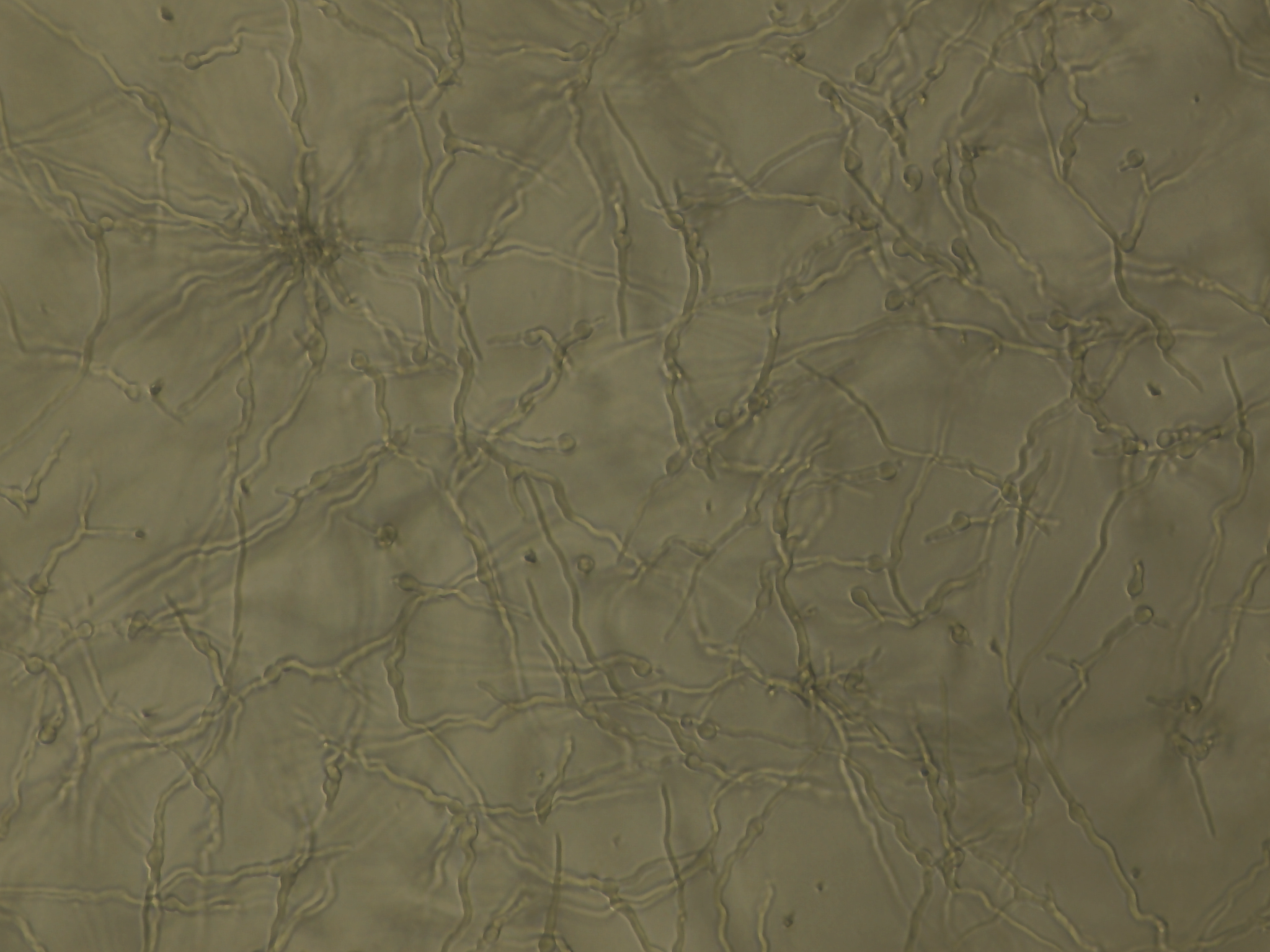

Supplement: Supplementary file 5 — Source data Fig. 3 [file 44321_2025_340_MOESM5_ESM.zip › Figure 3/Figure 3E/300724 cea10 pds 0.3.tif]

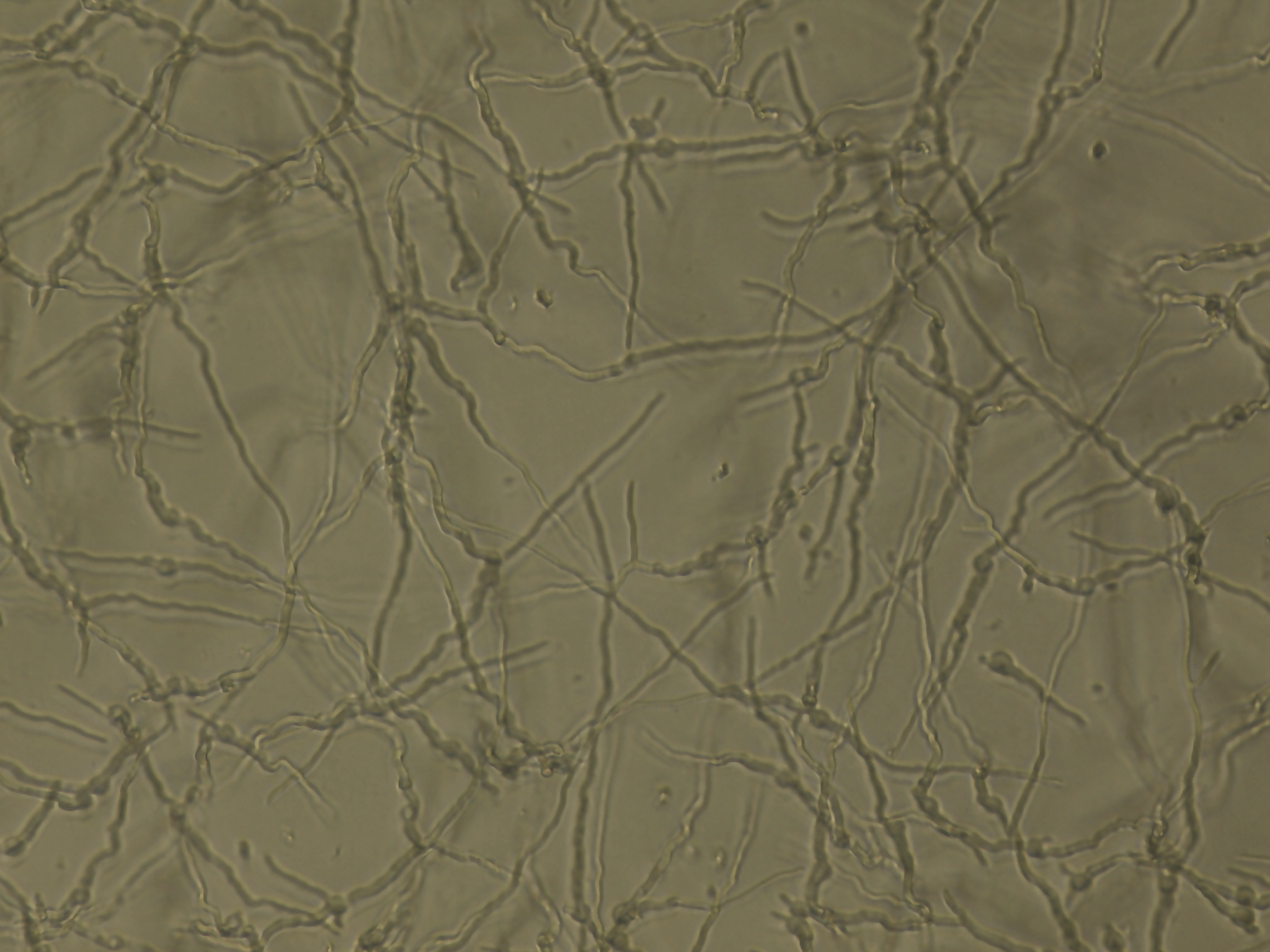

Supplement: Supplementary file 5 — Source data Fig. 3 [file 44321_2025_340_MOESM5_ESM.zip › Figure 3/Figure 3E/300724 cea10 pds 1.5.tif]

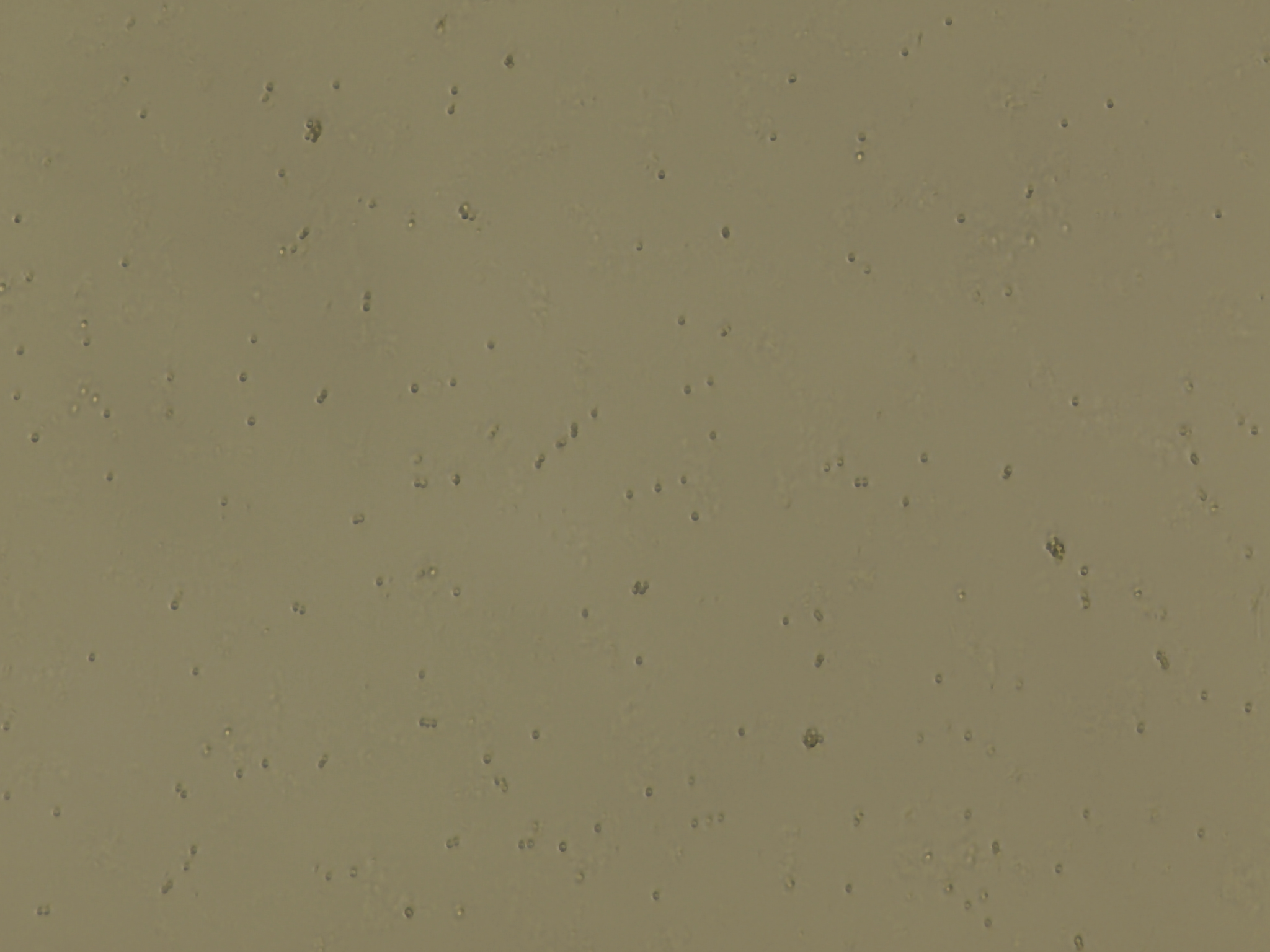

Supplement: Supplementary file 5 — Source data Fig. 3 [file 44321_2025_340_MOESM5_ESM.zip › Figure 3/Figure 3E/300724 cea10 pds 12.5.tif]

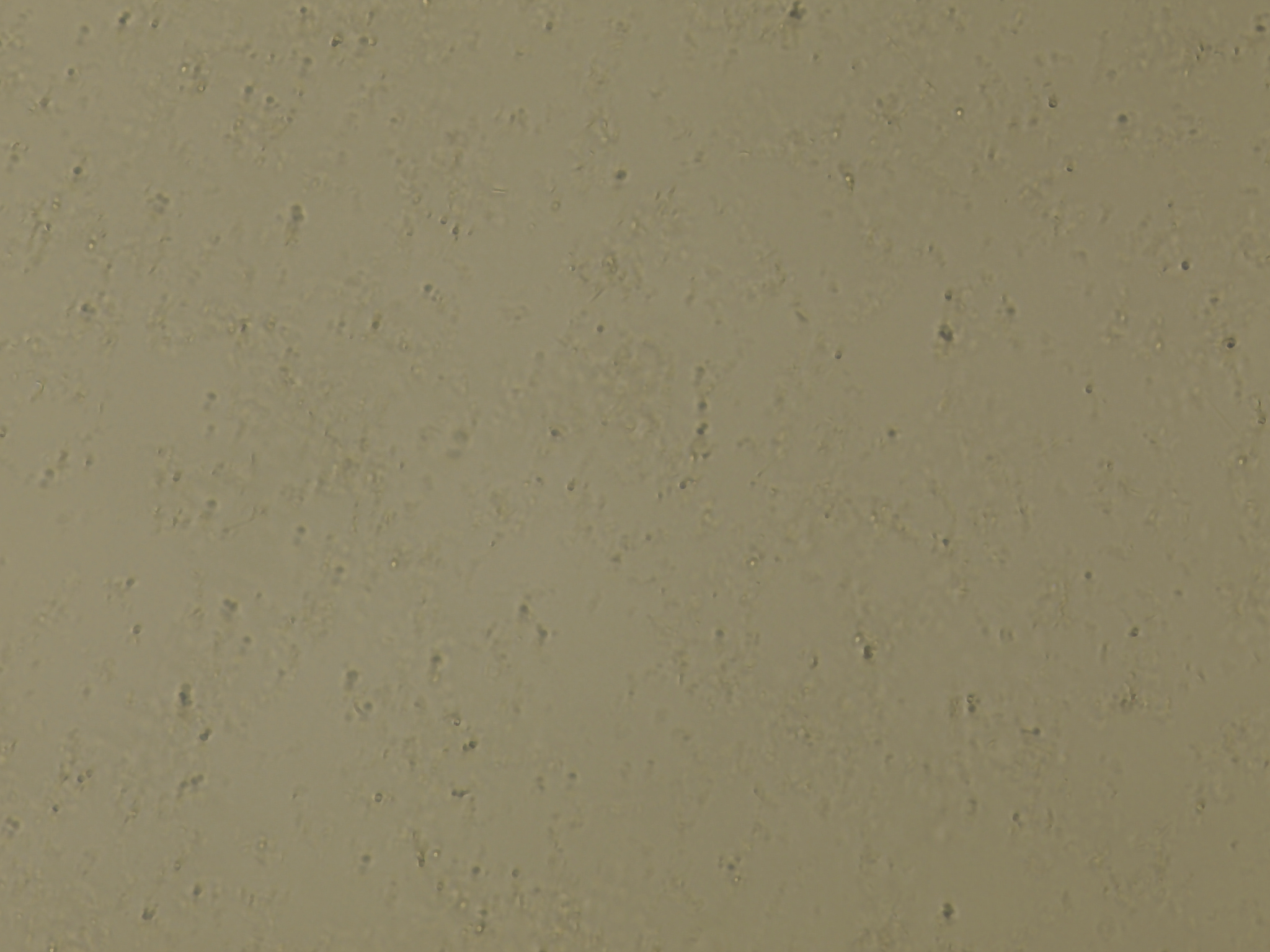

Supplement: Supplementary file 5 — Source data Fig. 3 [file 44321_2025_340_MOESM5_ESM.zip › Figure 3/Figure 3E/300724 cea10 pds 25.tif]

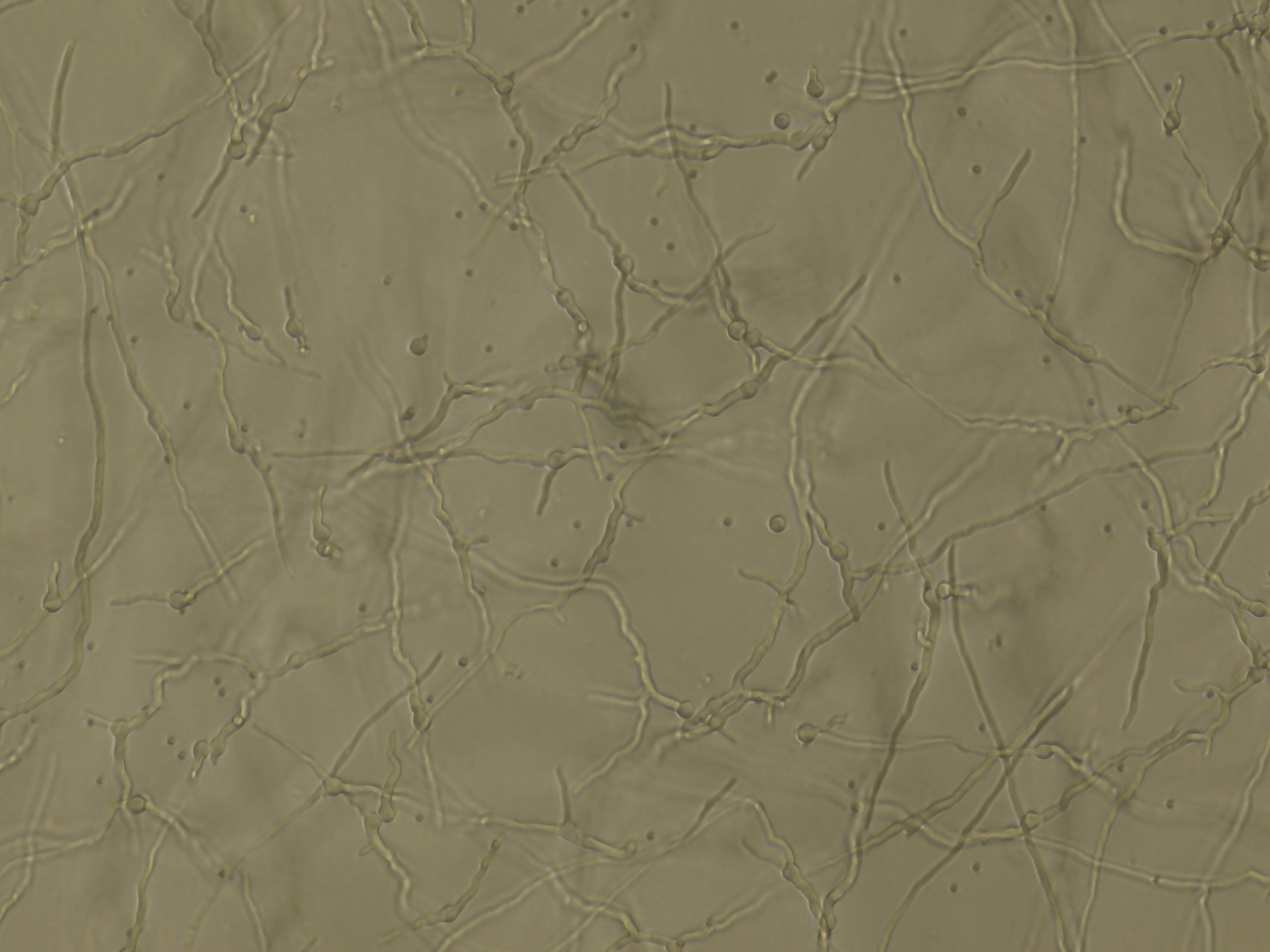

Supplement: Supplementary file 5 — Source data Fig. 3 [file 44321_2025_340_MOESM5_ESM.zip › Figure 3/Figure 3E/300724 cea10 pds 3.1.tif]

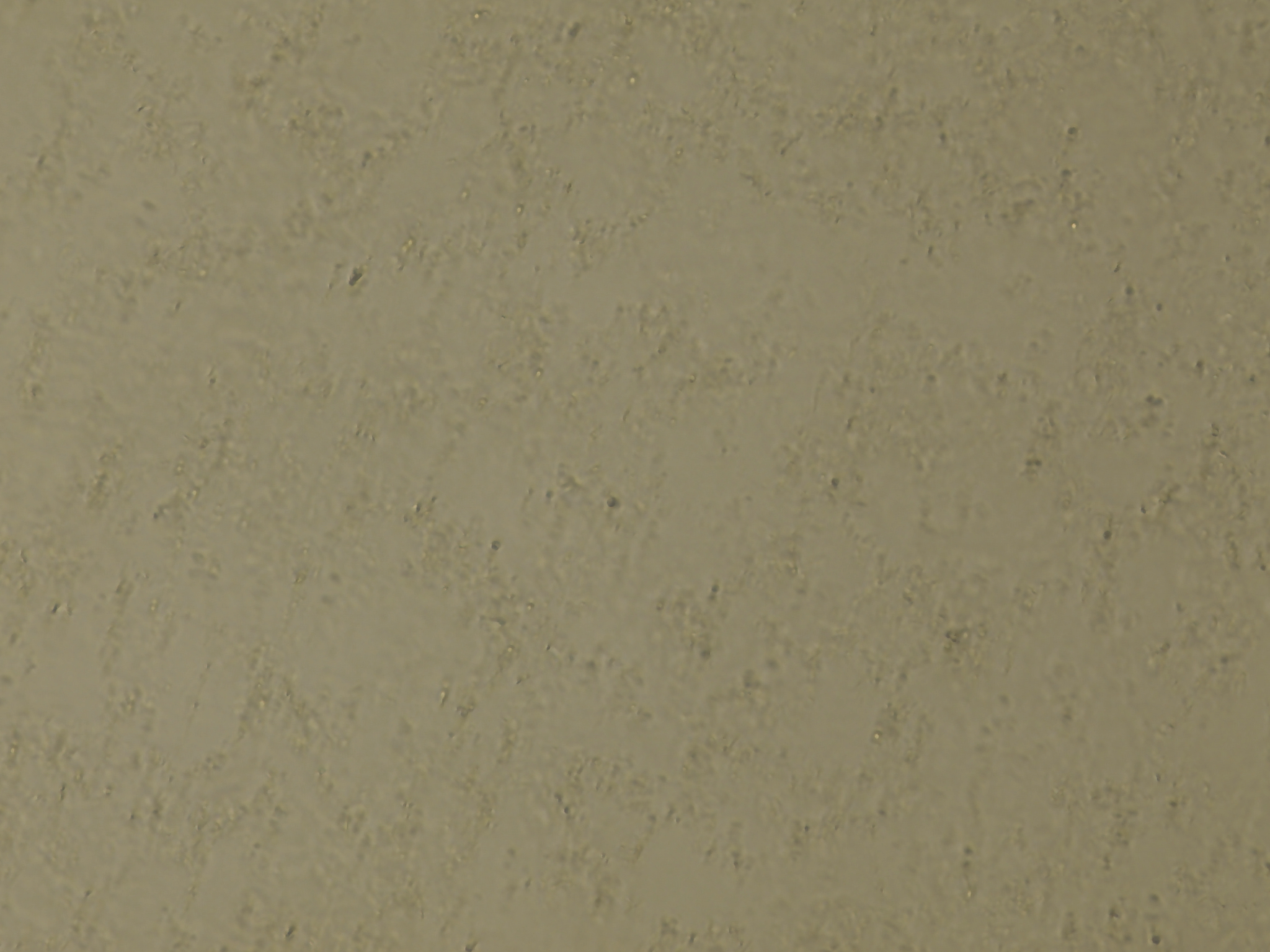

Supplement: Supplementary file 5 — Source data Fig. 3 [file 44321_2025_340_MOESM5_ESM.zip › Figure 3/Figure 3E/300724 cea10 pds 50.tif]

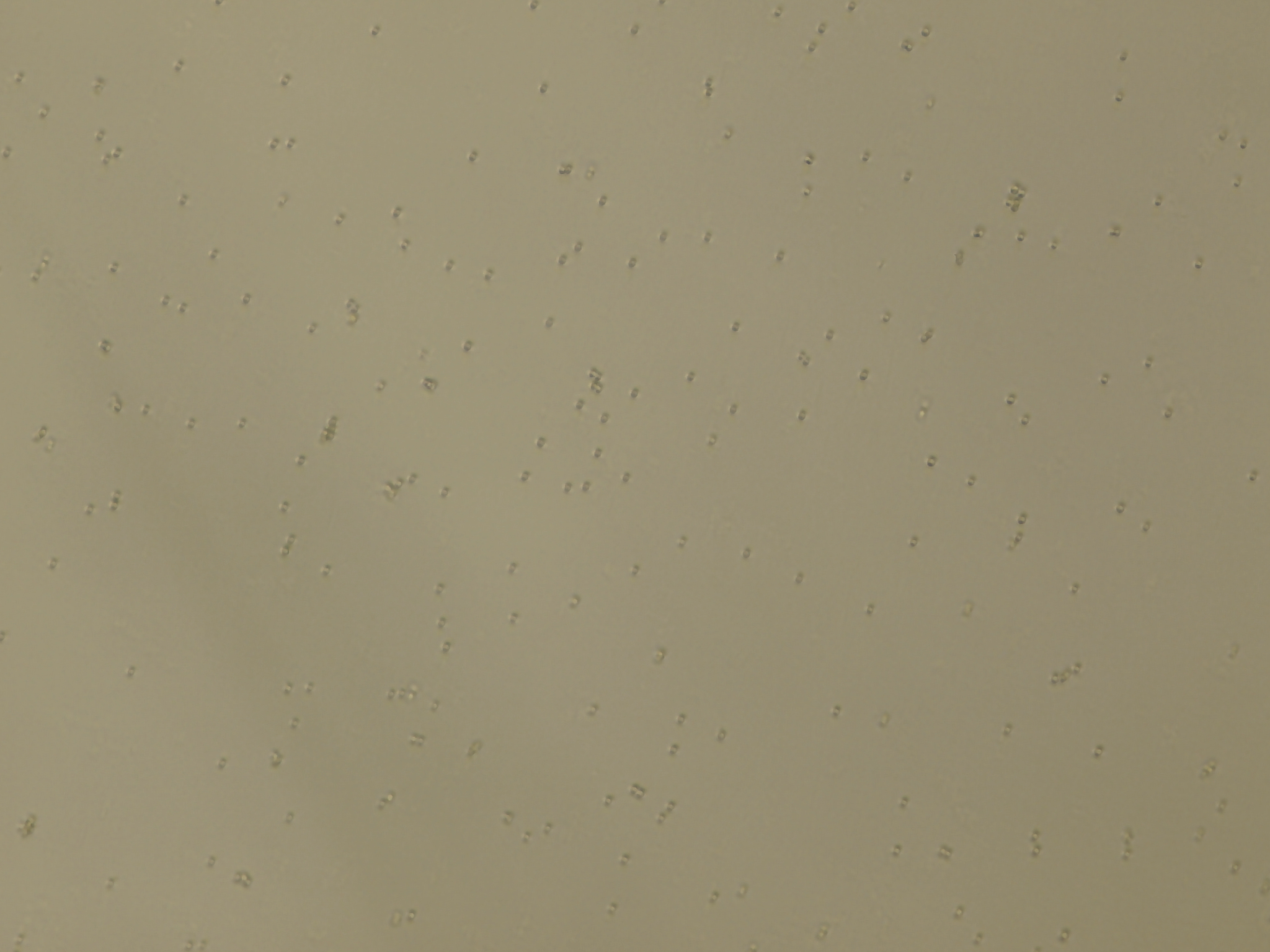

Supplement: Supplementary file 5 — Source data Fig. 3 [file 44321_2025_340_MOESM5_ESM.zip › Figure 3/Figure 3E/300724 cea10 pds 6.25.tif]

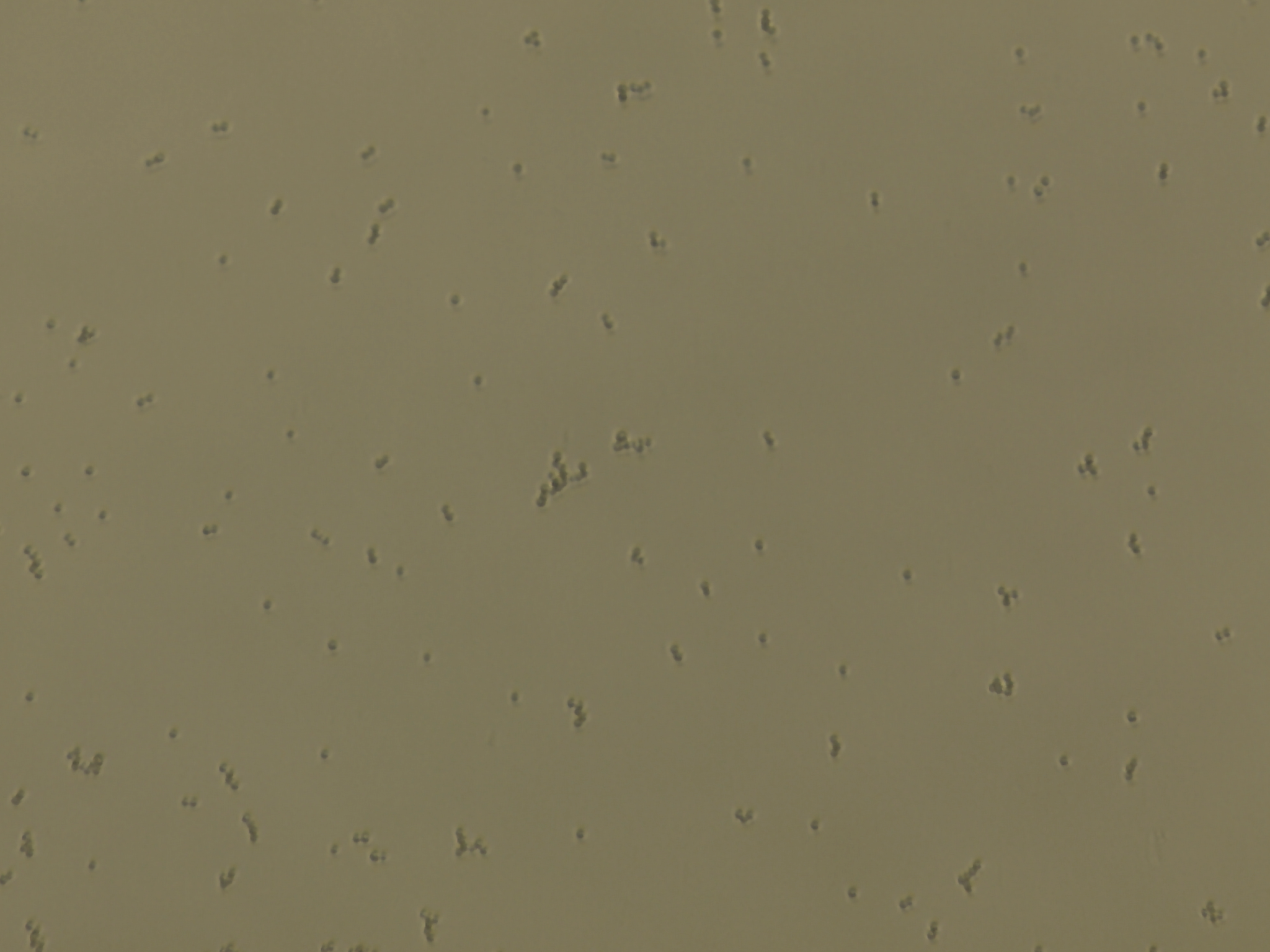

Supplement: Supplementary file 5 — Source data Fig. 3 [file 44321_2025_340_MOESM5_ESM.zip › Figure 3/Figure 3E/300724 cea10 pds neg.tif]

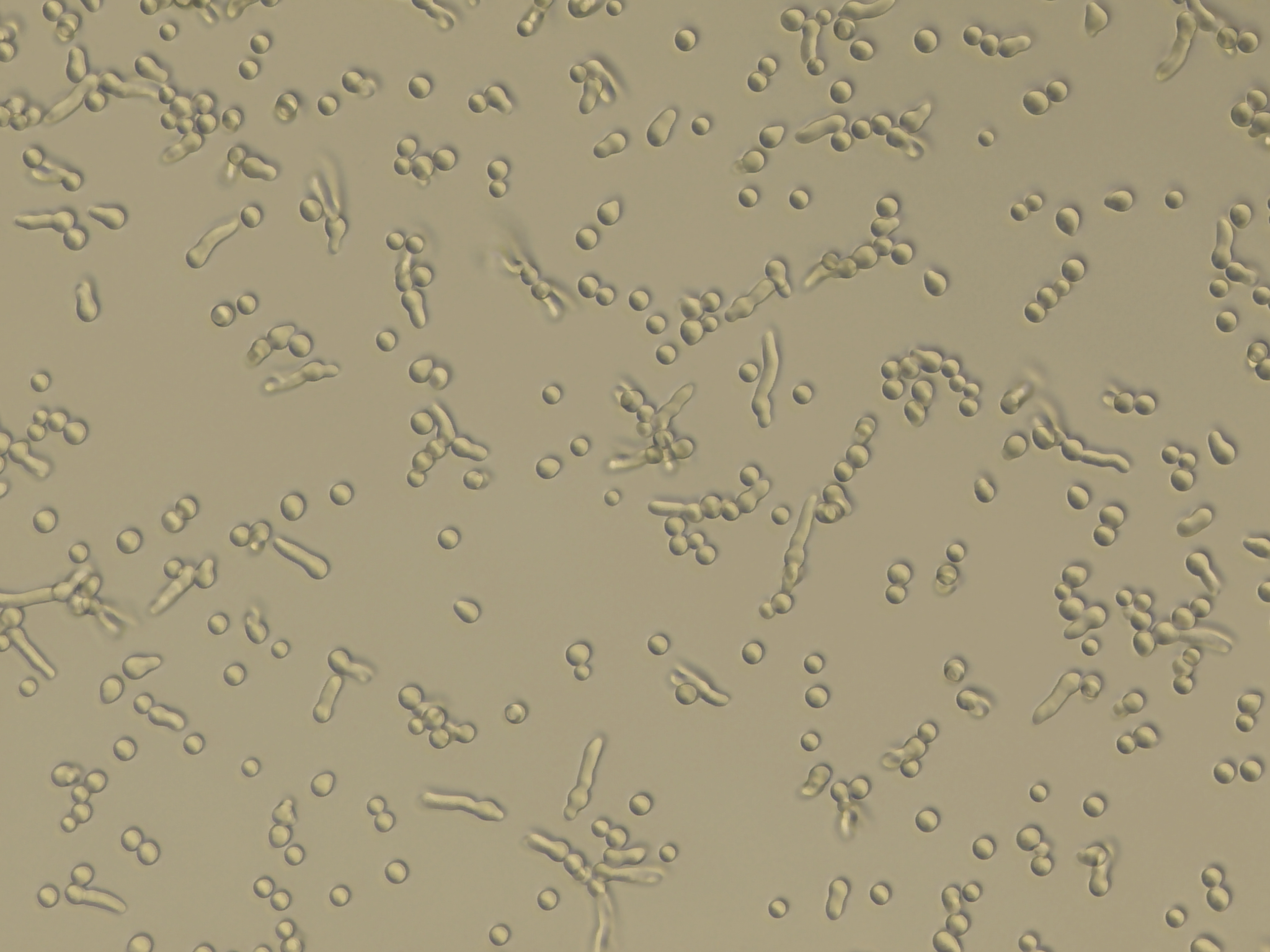

Supplement: Supplementary file 5 — Source data Fig. 3 [file 44321_2025_340_MOESM5_ESM.zip › Figure 3/Figure 3E/300724 cea10 phen 0.3.tif]

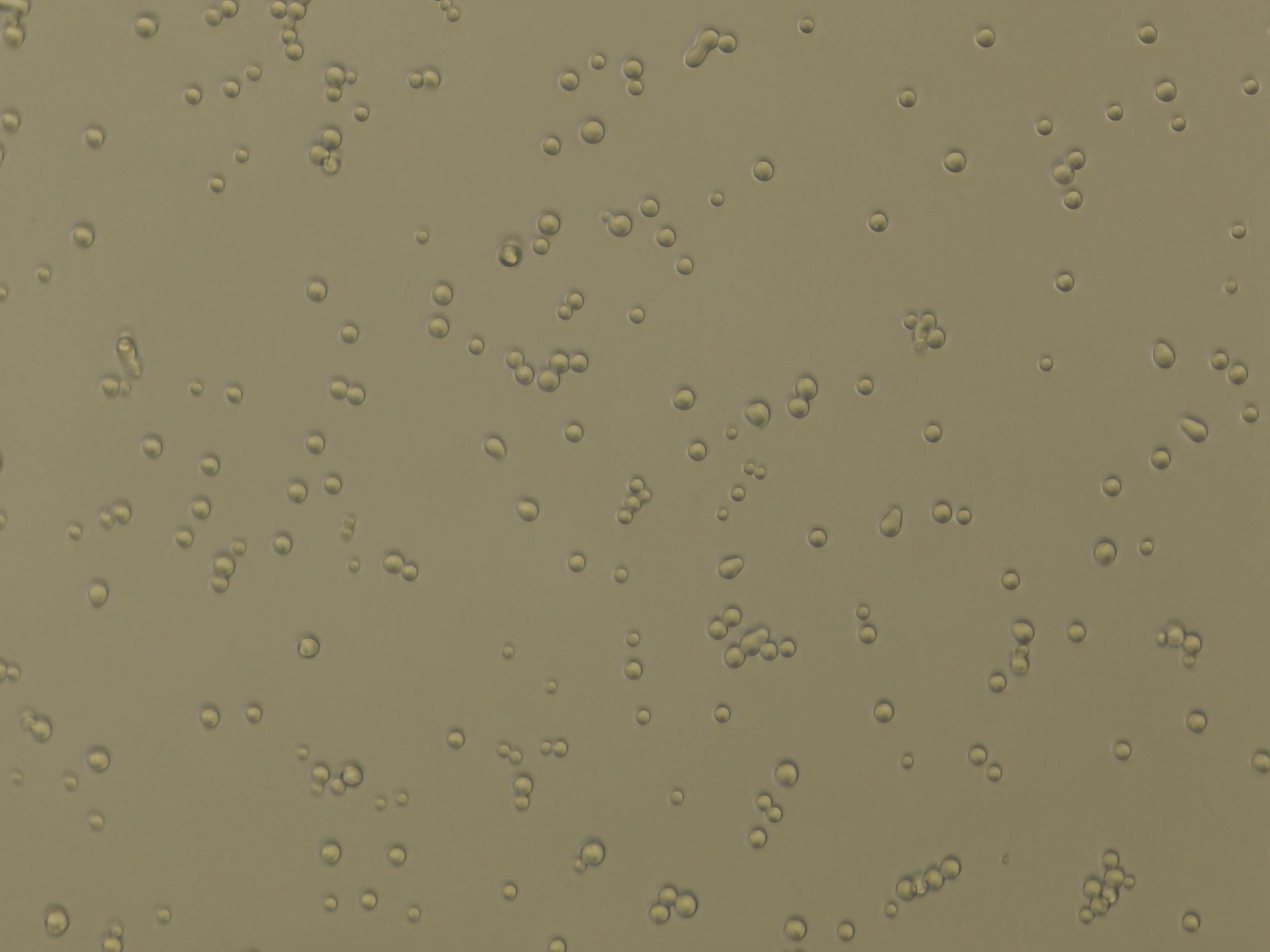

Supplement: Supplementary file 5 — Source data Fig. 3 [file 44321_2025_340_MOESM5_ESM.zip › Figure 3/Figure 3E/300724 cea10 phen 0.7.tif]

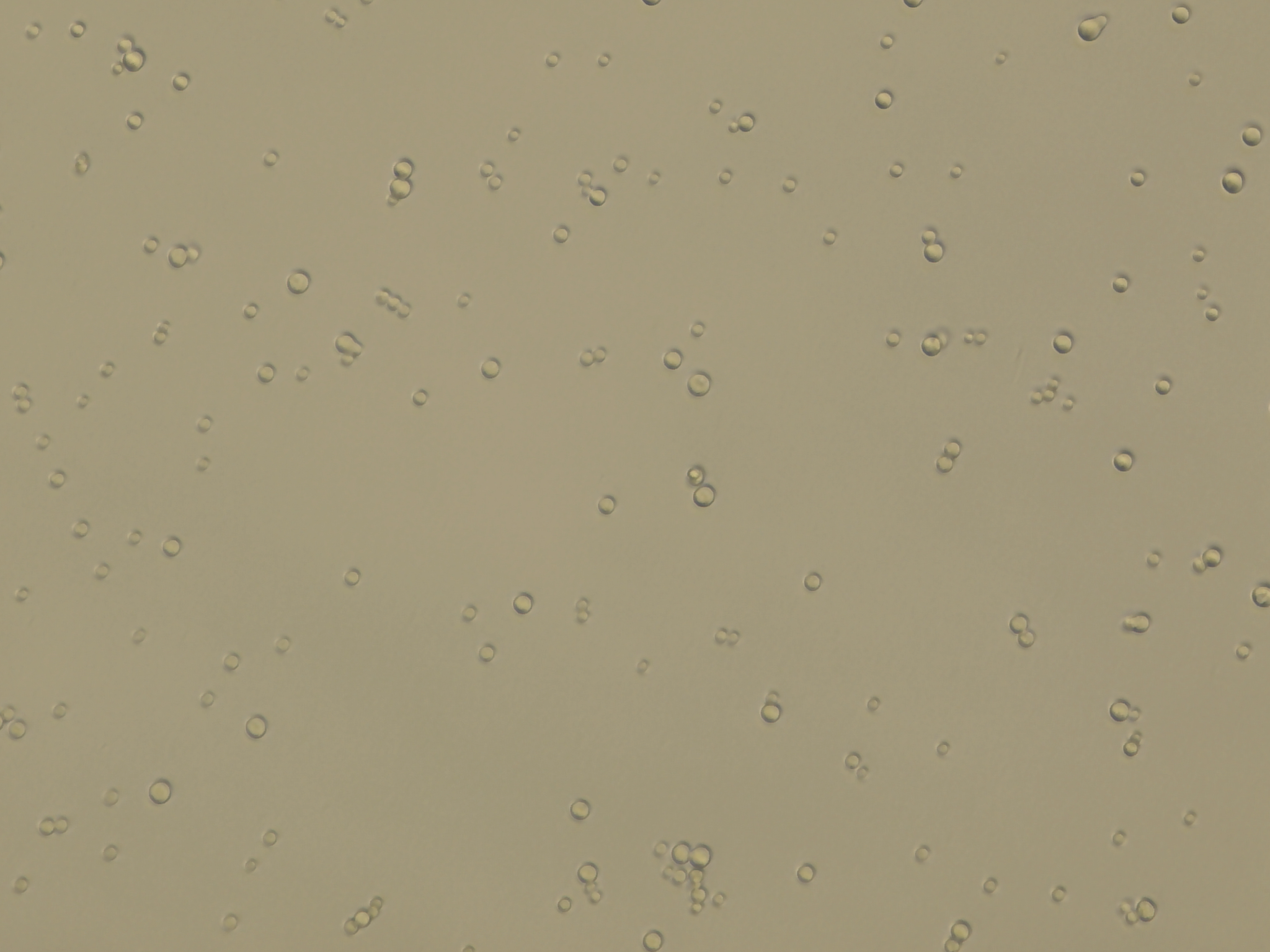

Supplement: Supplementary file 5 — Source data Fig. 3 [file 44321_2025_340_MOESM5_ESM.zip › Figure 3/Figure 3E/300724 cea10 phen 1.5.tif]

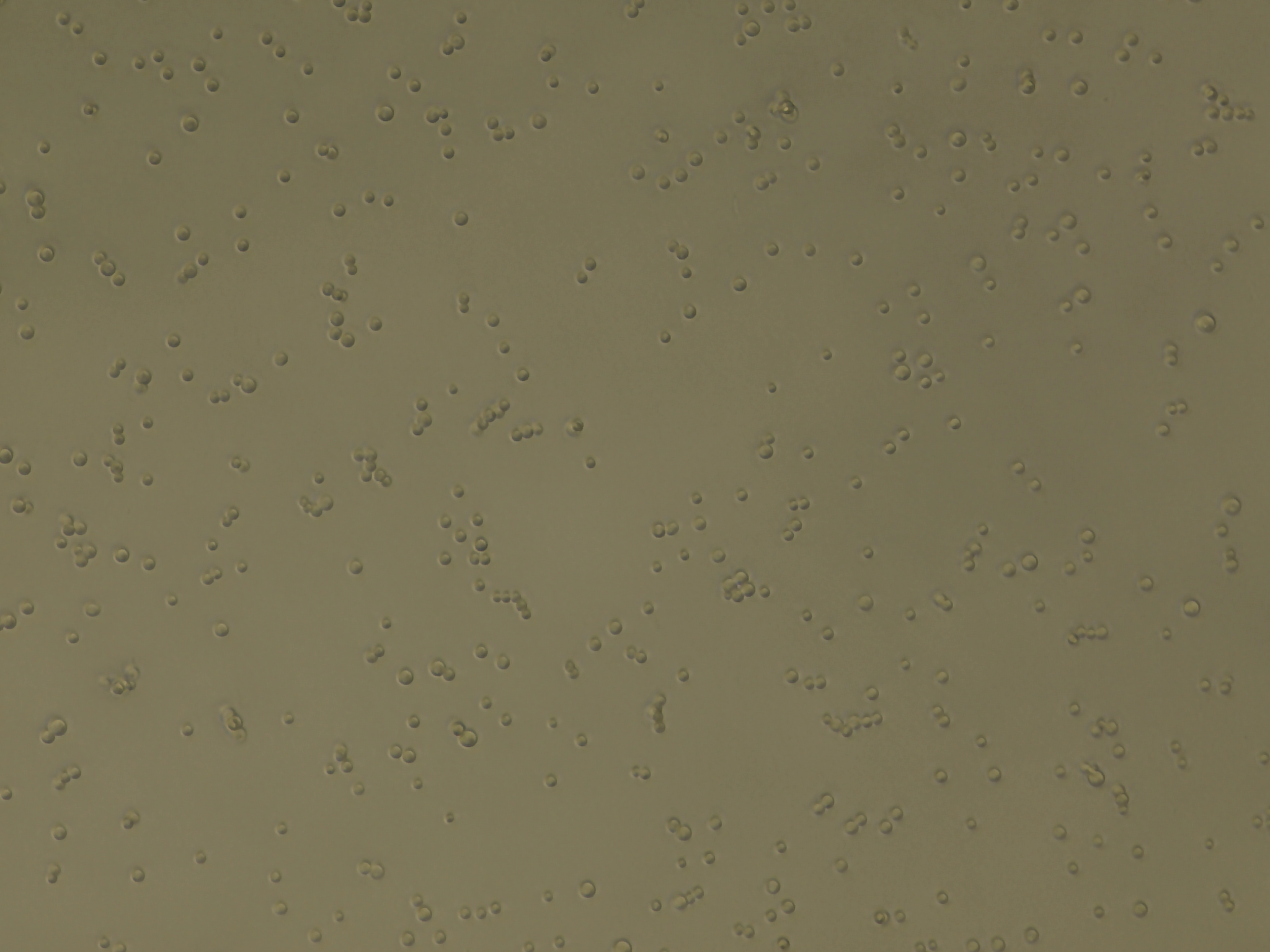

Supplement: Supplementary file 5 — Source data Fig. 3 [file 44321_2025_340_MOESM5_ESM.zip › Figure 3/Figure 3E/300724 cea10 phen 12.5.tif]

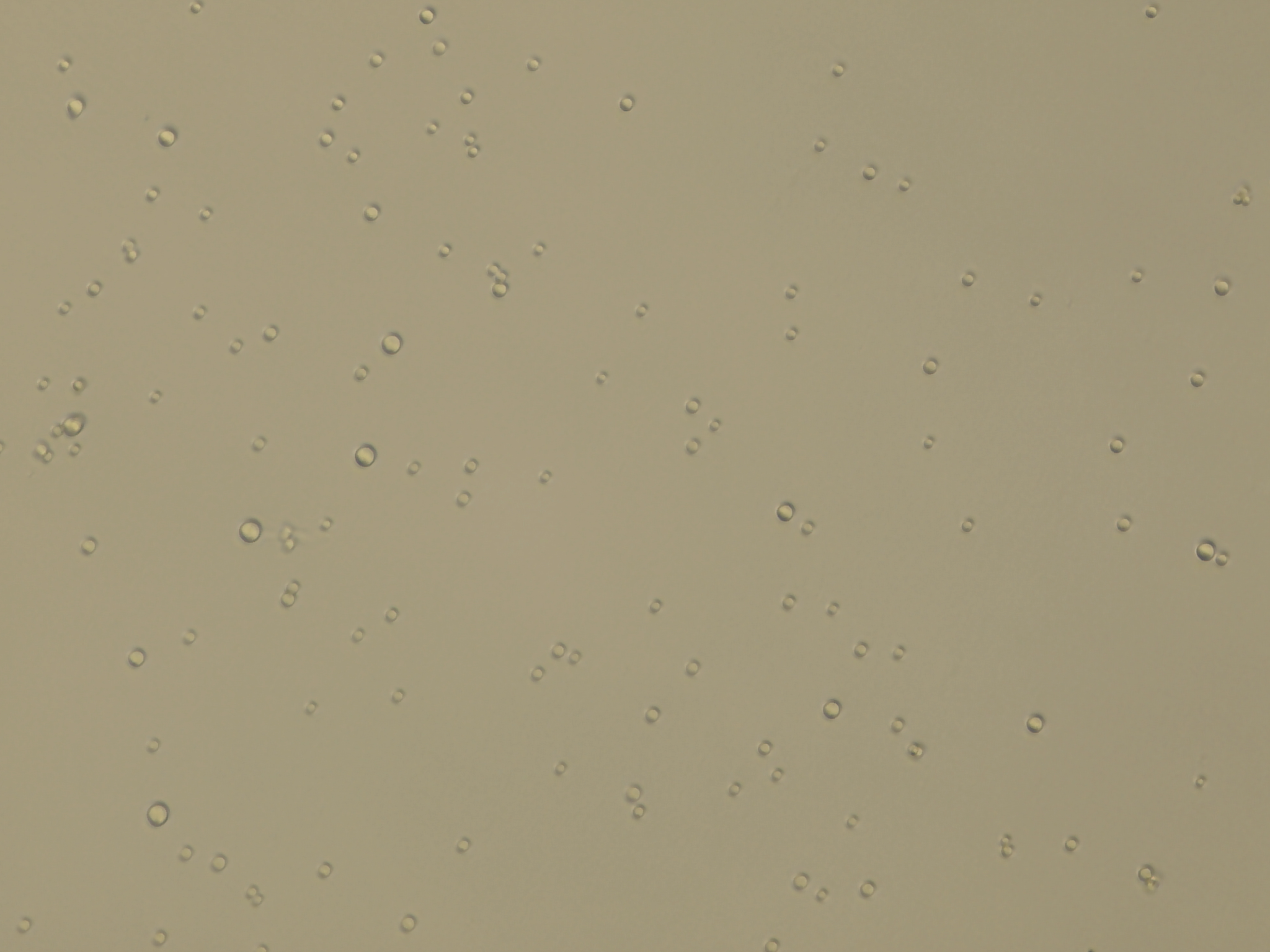

Supplement: Supplementary file 5 — Source data Fig. 3 [file 44321_2025_340_MOESM5_ESM.zip › Figure 3/Figure 3E/300724 cea10 phen 3.1.tif]

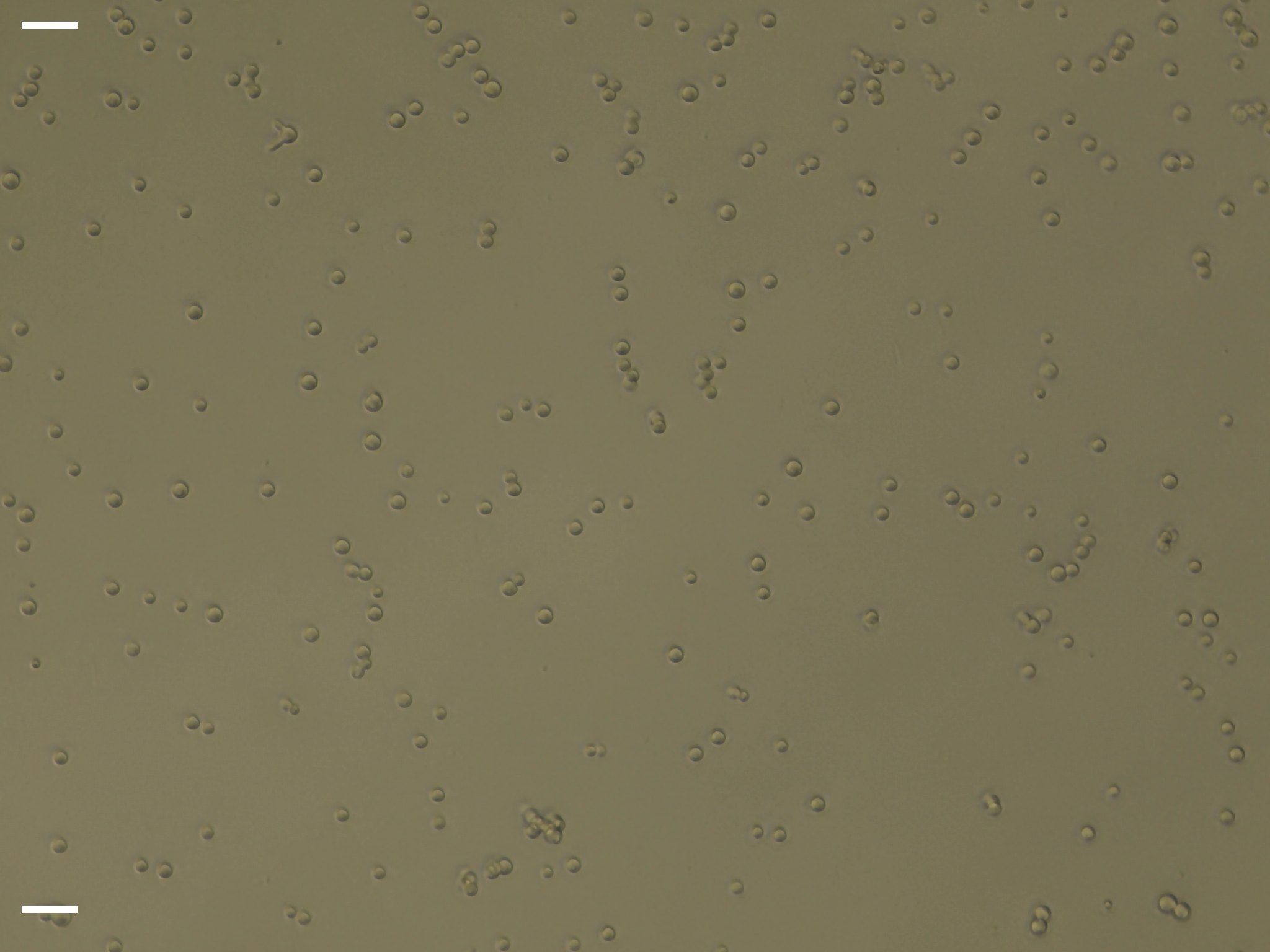

Supplement: Supplementary file 5 — Source data Fig. 3 [file 44321_2025_340_MOESM5_ESM.zip › Figure 3/Figure 3E/300724 cea10 phen 50 SCALE.tif]

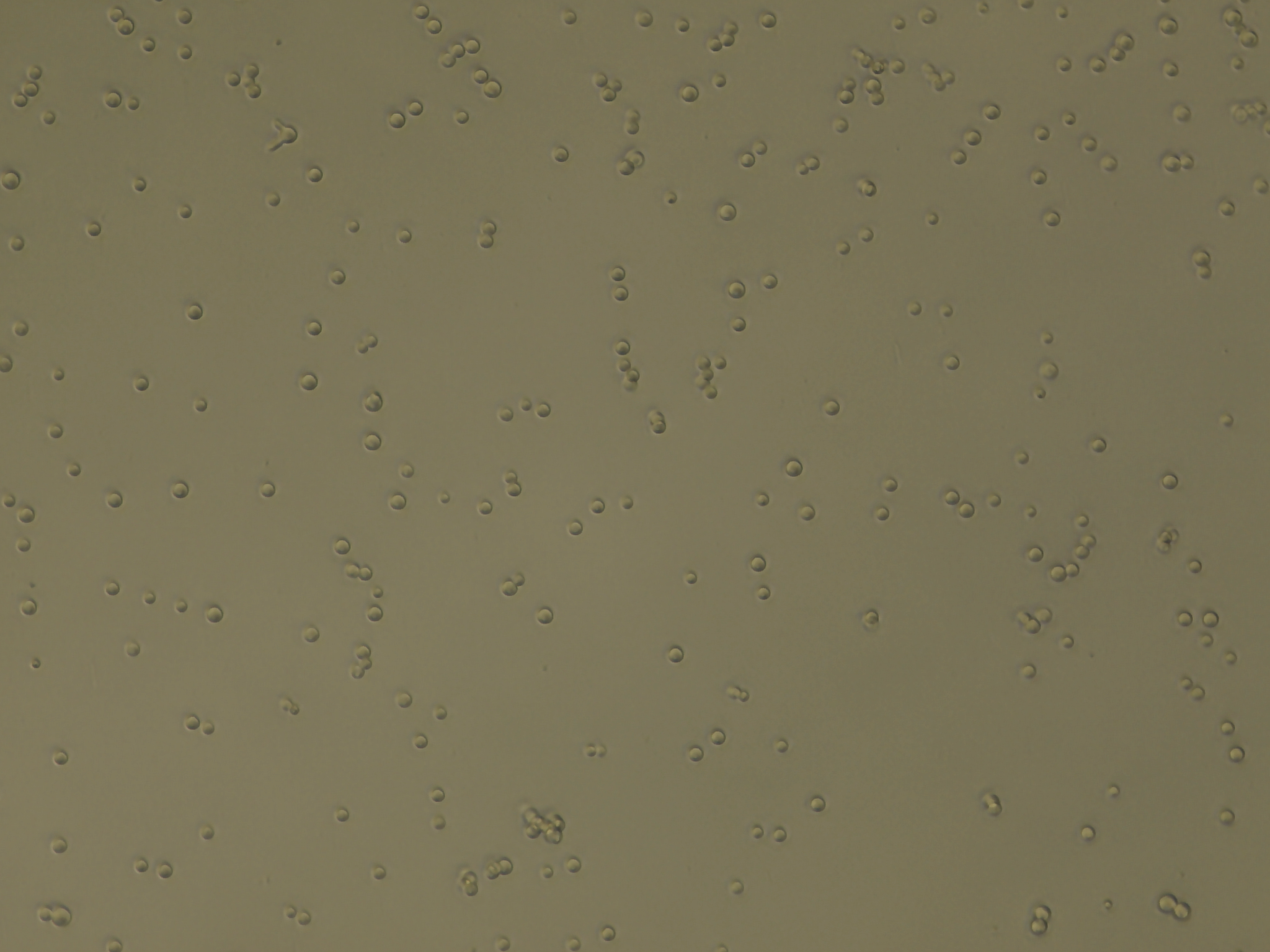

Supplement: Supplementary file 5 — Source data Fig. 3 [file 44321_2025_340_MOESM5_ESM.zip › Figure 3/Figure 3E/300724 cea10 phen 50.tif]

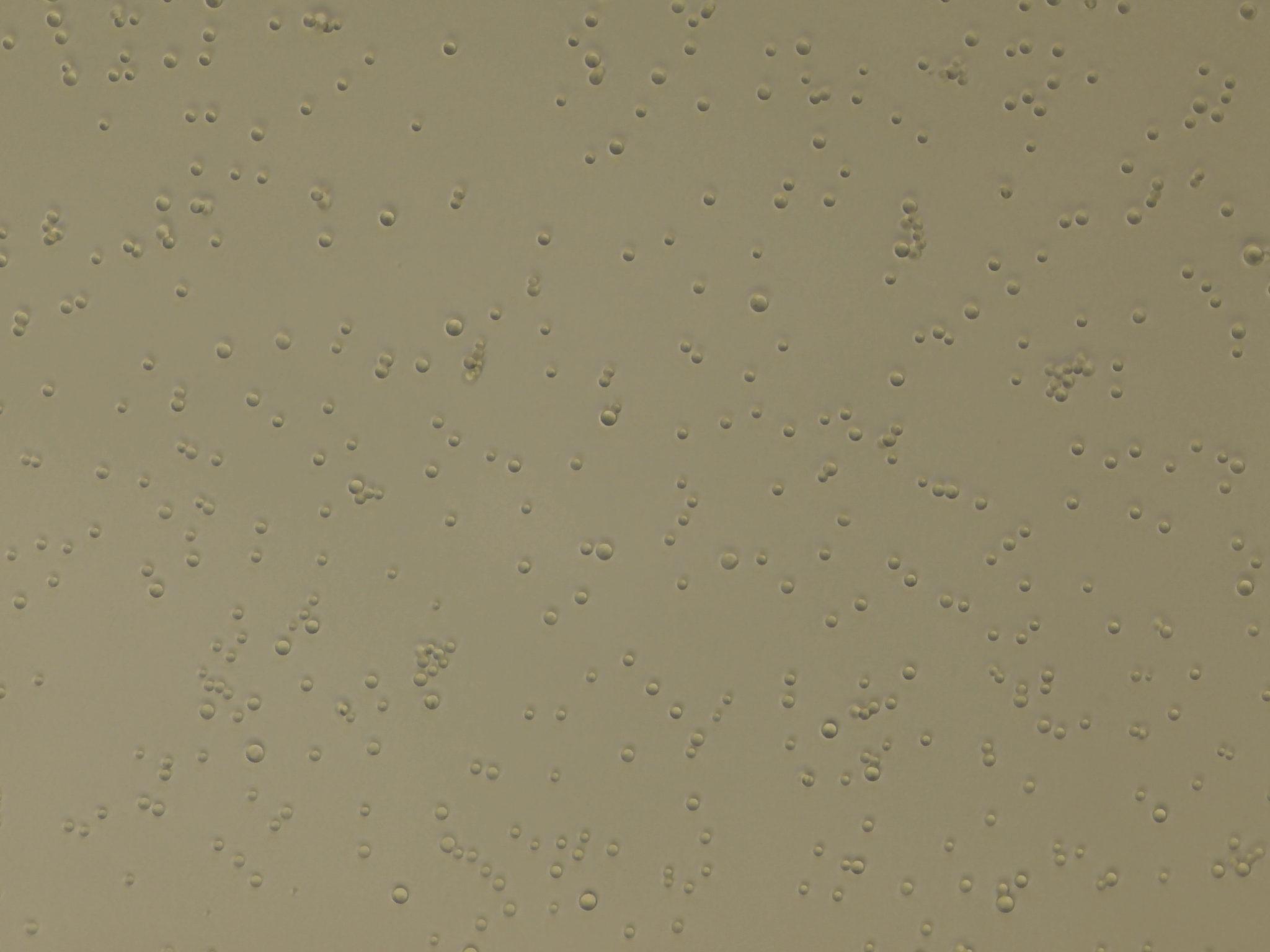

Supplement: Supplementary file 5 — Source data Fig. 3 [file 44321_2025_340_MOESM5_ESM.zip › Figure 3/Figure 3E/300724 cea10 phen 6.25.tif]

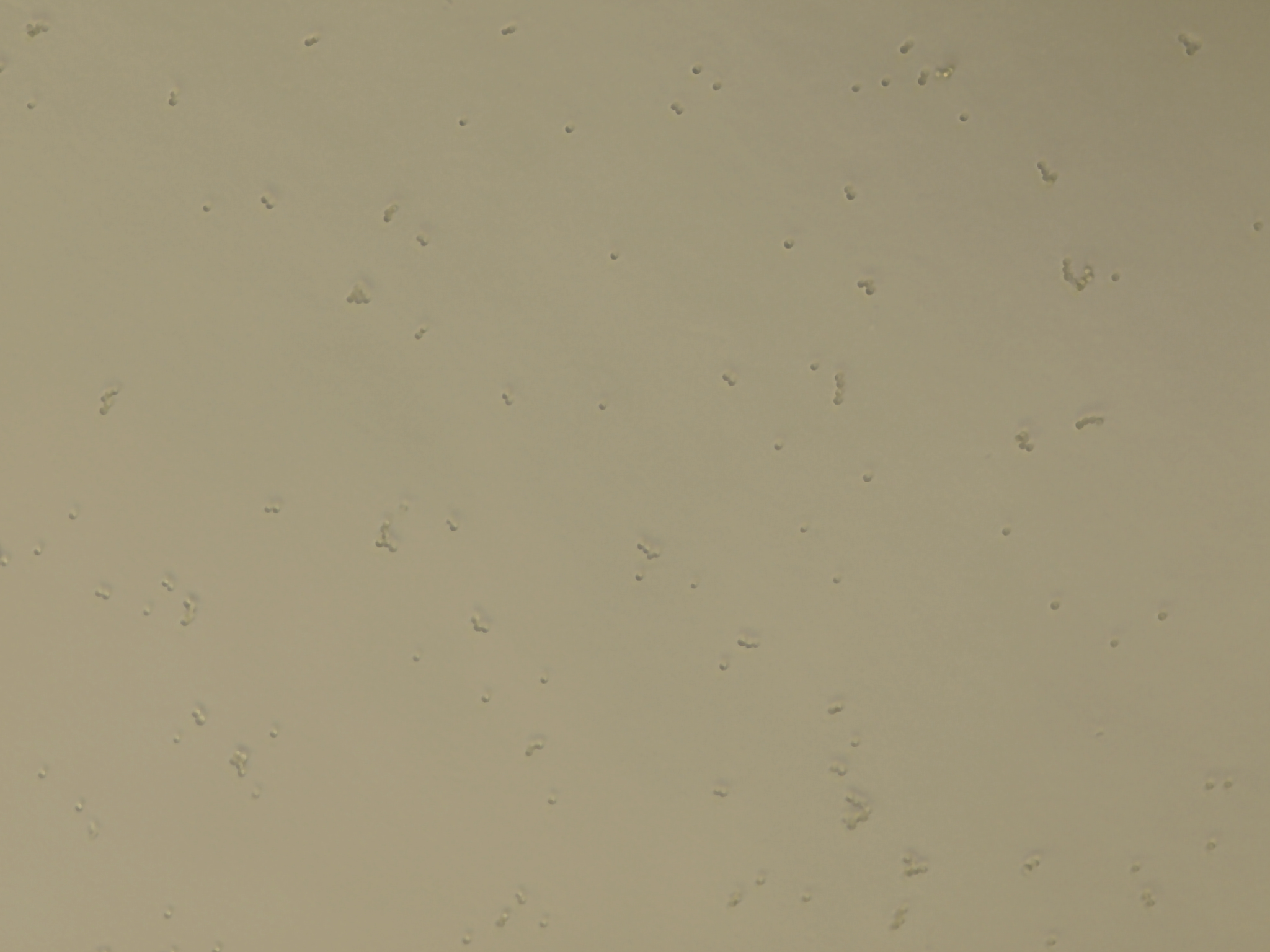

Supplement: Supplementary file 5 — Source data Fig. 3 [file 44321_2025_340_MOESM5_ESM.zip › Figure 3/Figure 3E/300724 cea10 phen neg.tif]

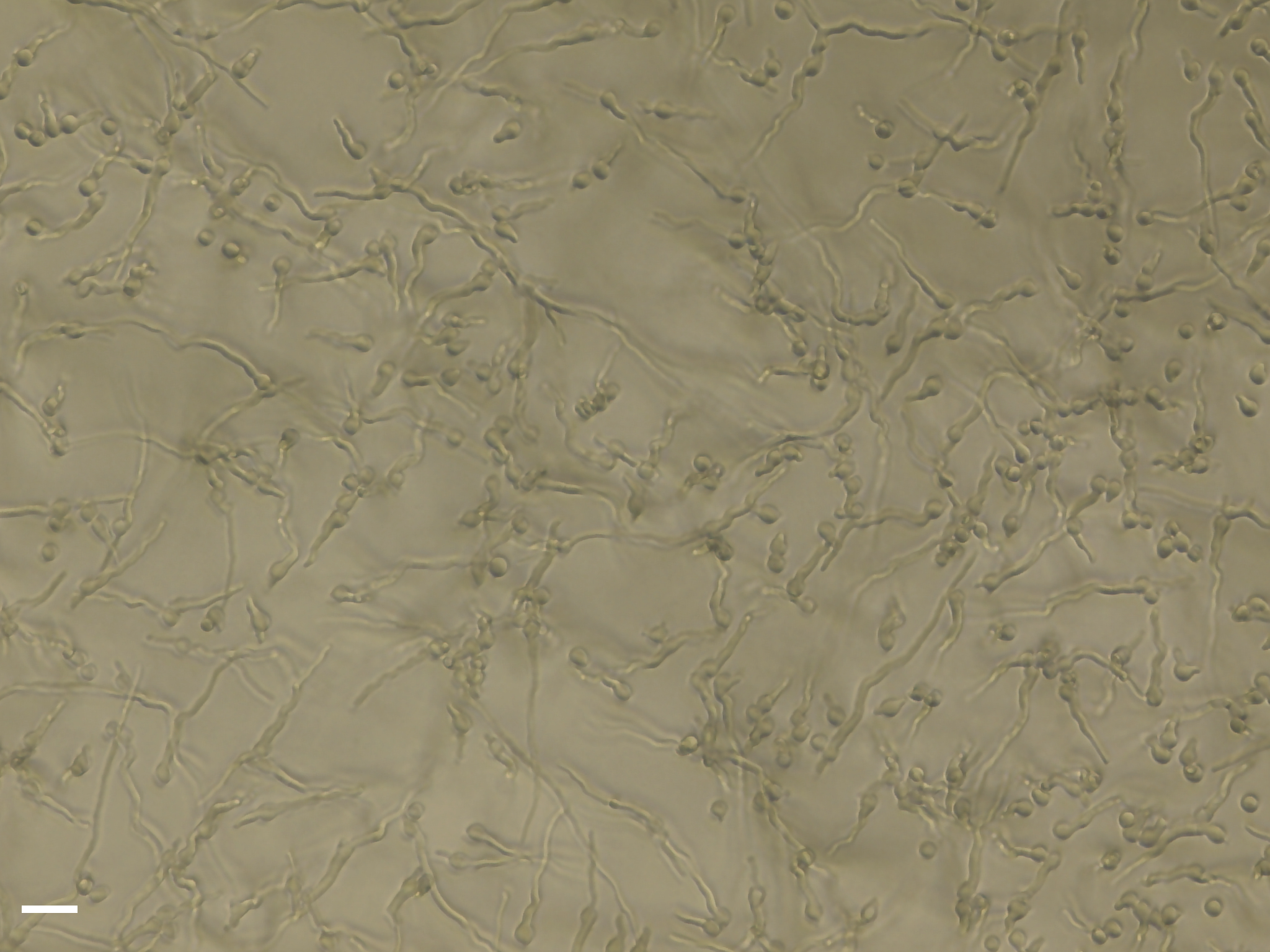

Supplement: Supplementary file 5 — Source data Fig. 3 [file 44321_2025_340_MOESM5_ESM.zip › Figure 3/Figure 3E/300724 cea10 phen pos SCALE.tif]

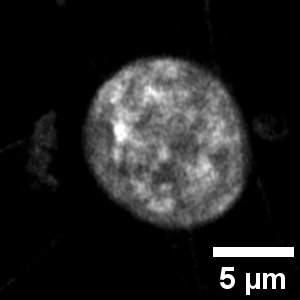

Supplement: Supplementary file 7 — Source data Fig. 5 [file 44321_2025_340_MOESM7_ESM.zip › Figure 5/Figure 5C/DNA Damage Images/CONTROL/Composite DAPI CONTROL SCALEBAR ZOOM.jpg]

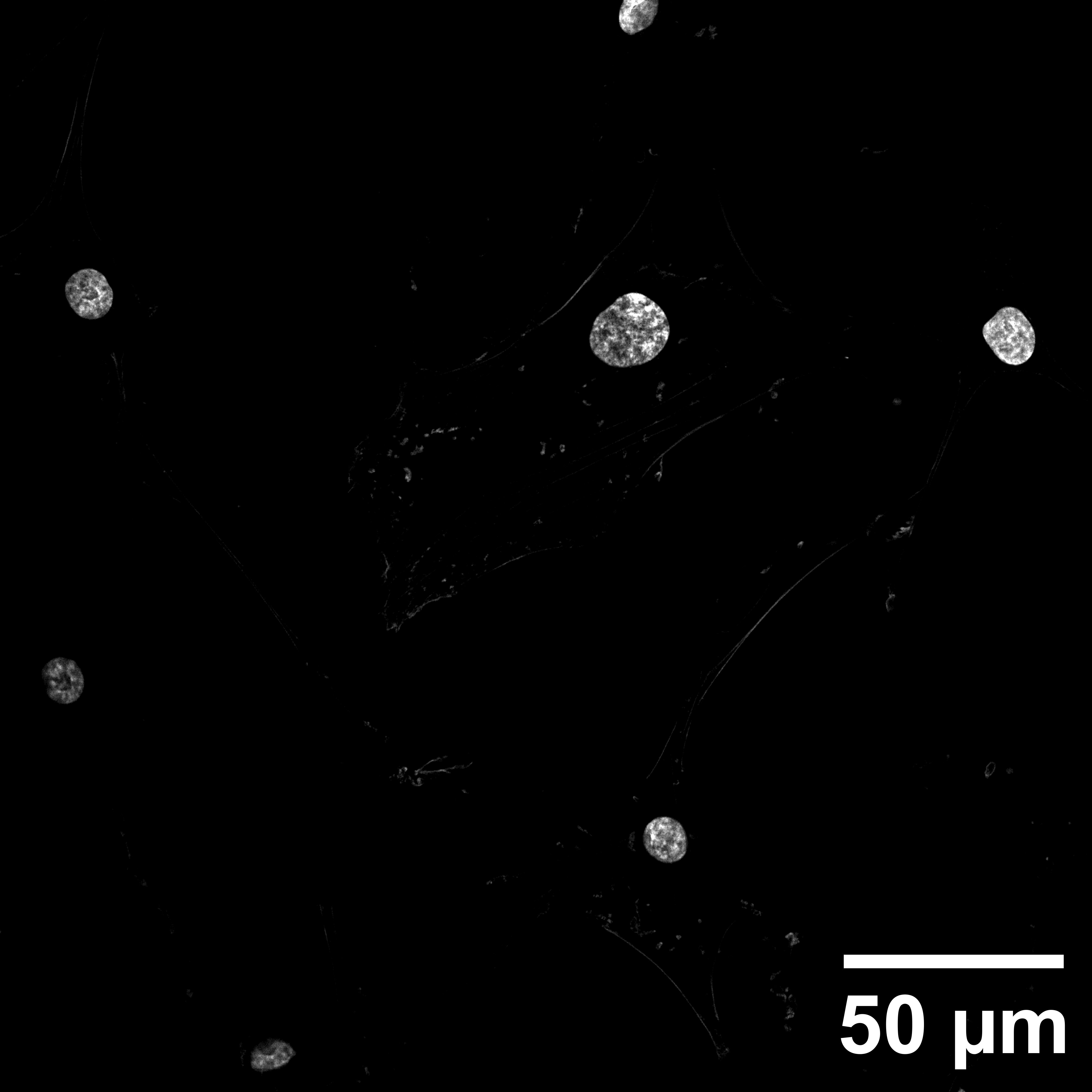

Supplement: Supplementary file 7 — Source data Fig. 5 [file 44321_2025_340_MOESM7_ESM.zip › Figure 5/Figure 5C/DNA Damage Images/CONTROL/Composite DAPI CONTROL SCALEBAR.jpg]

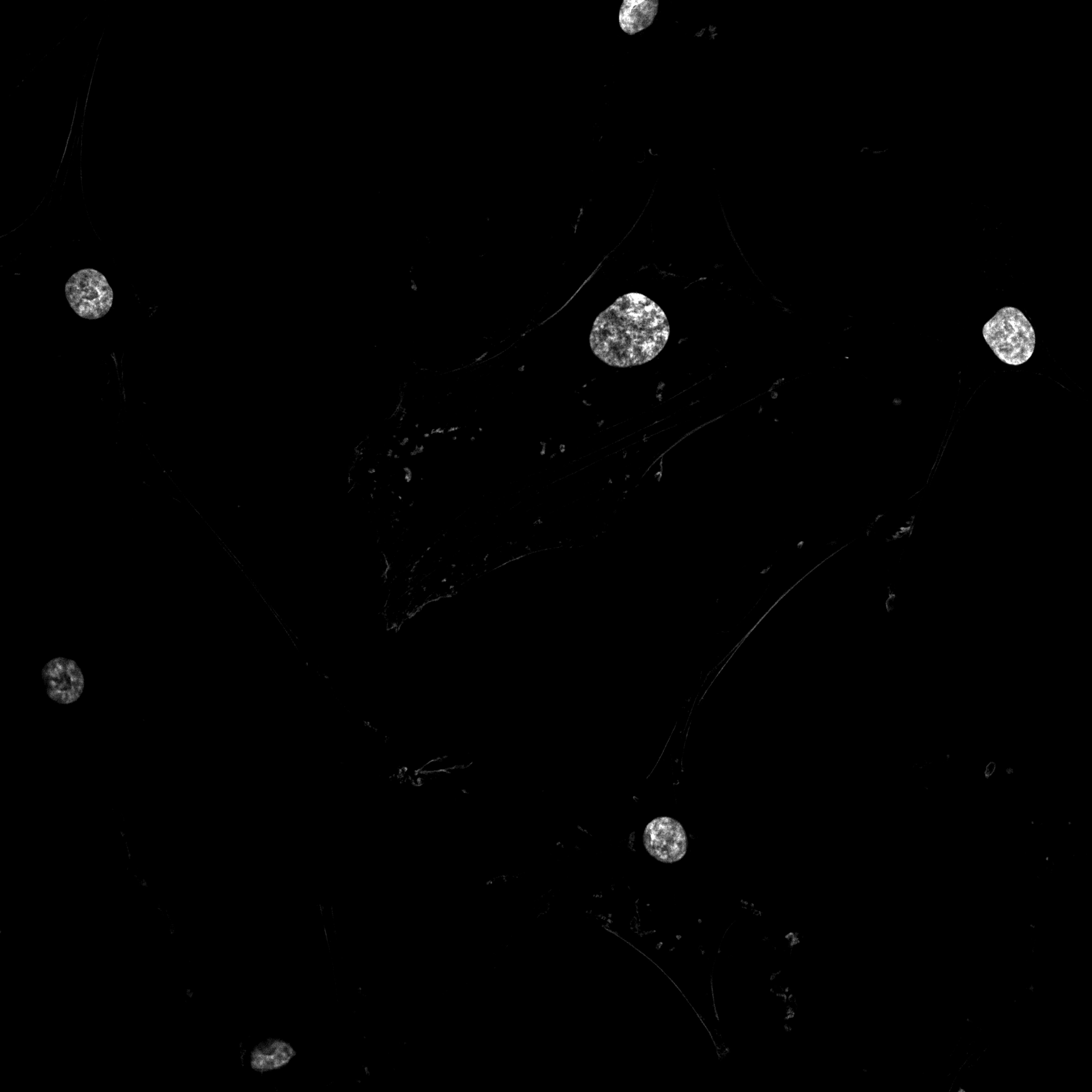

Supplement: Supplementary file 7 — Source data Fig. 5 [file 44321_2025_340_MOESM7_ESM.zip › Figure 5/Figure 5C/DNA Damage Images/CONTROL/Composite DAPI CONTROL.jpg]

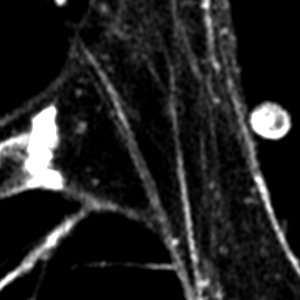

Supplement: Supplementary file 7 — Source data Fig. 5 [file 44321_2025_340_MOESM7_ESM.zip › Figure 5/Figure 5C/DNA Damage Images/CONTROL/Composite F ACTIN CONTROL ZOOM.jpg]

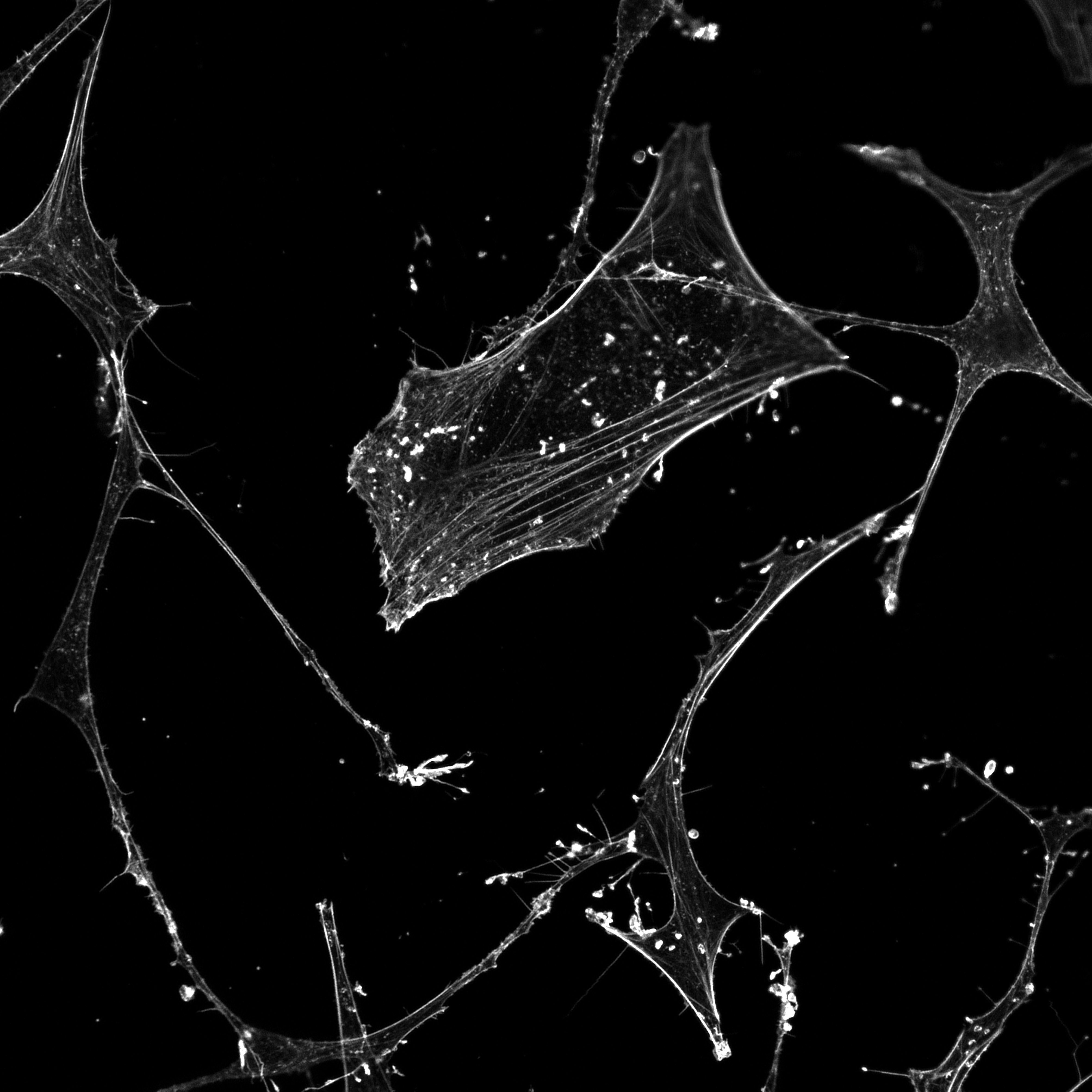

Supplement: Supplementary file 7 — Source data Fig. 5 [file 44321_2025_340_MOESM7_ESM.zip › Figure 5/Figure 5C/DNA Damage Images/CONTROL/Composite F ACTIN CONTROL.jpg]

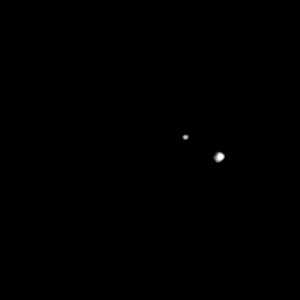

Supplement: Supplementary file 7 — Source data Fig. 5 [file 44321_2025_340_MOESM7_ESM.zip › Figure 5/Figure 5C/DNA Damage Images/CONTROL/Composite H2AX CONTROL ZOOM.jpg]

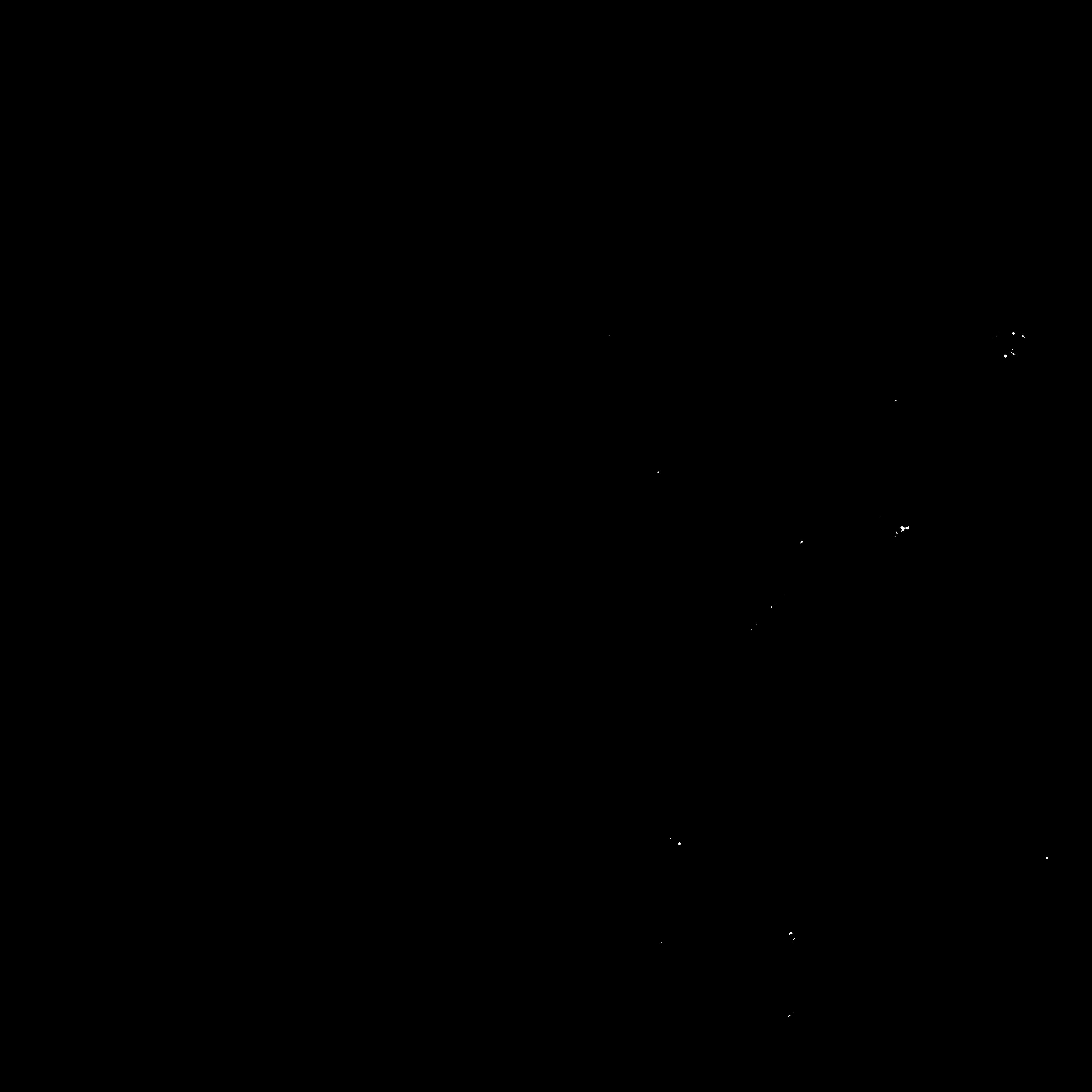

Supplement: Supplementary file 7 — Source data Fig. 5 [file 44321_2025_340_MOESM7_ESM.zip › Figure 5/Figure 5C/DNA Damage Images/CONTROL/Composite H2AX CONTROL.jpg]

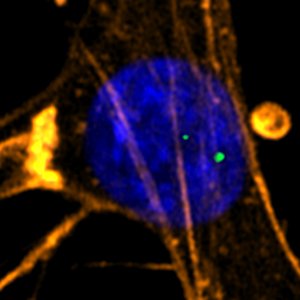

Supplement: Supplementary file 7 — Source data Fig. 5 [file 44321_2025_340_MOESM7_ESM.zip › Figure 5/Figure 5C/DNA Damage Images/CONTROL/Composite MERGED CONTROL ZOOM.jpg]

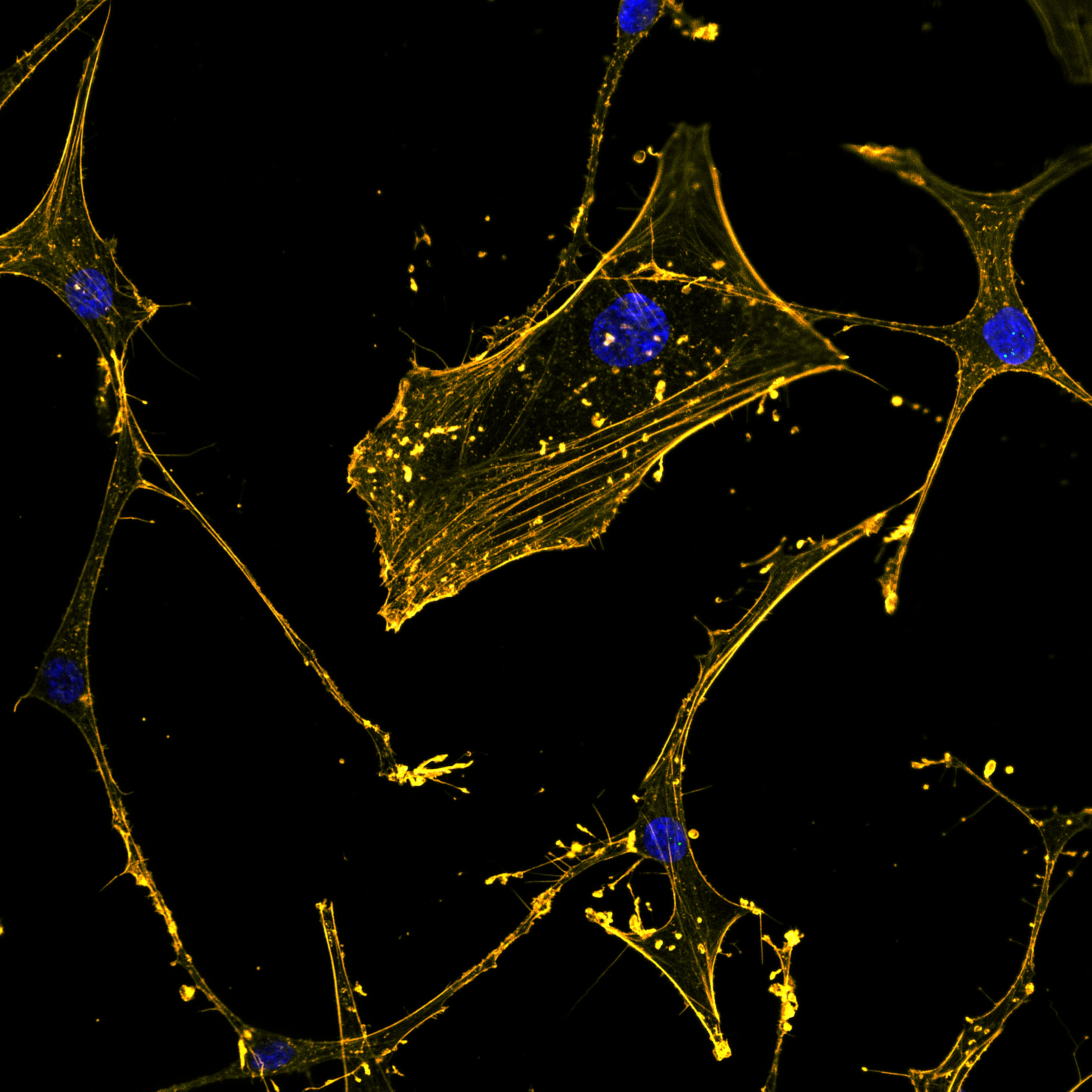

Supplement: Supplementary file 7 — Source data Fig. 5 [file 44321_2025_340_MOESM7_ESM.zip › Figure 5/Figure 5C/DNA Damage Images/CONTROL/Composite MERGED CONTROL.jpg]

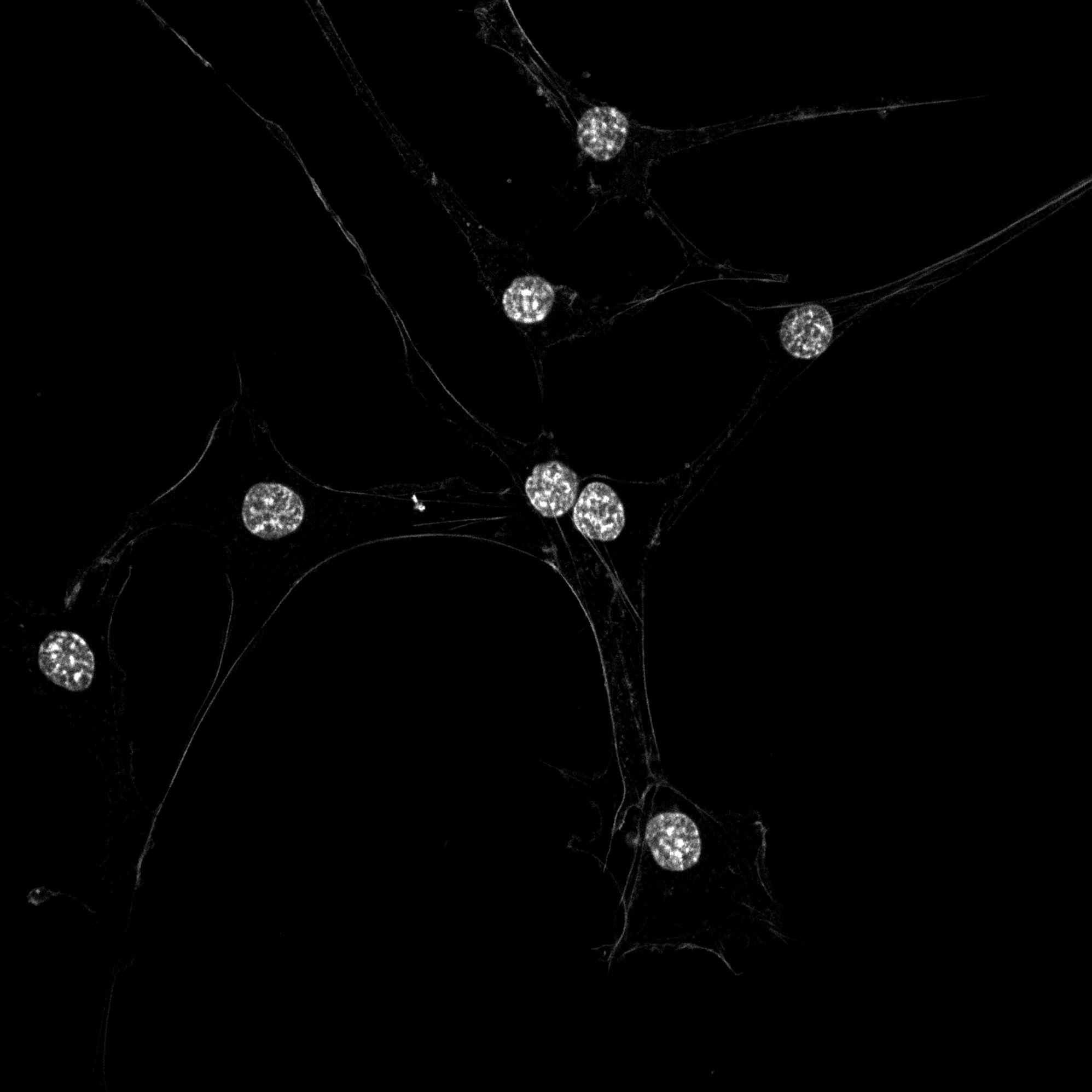

Supplement: Supplementary file 7 — Source data Fig. 5 [file 44321_2025_340_MOESM7_ESM.zip › Figure 5/Figure 5C/DNA Damage Images/PHENDC3 25/COMPOSITE DAPI PHENDC3 25.jpg]

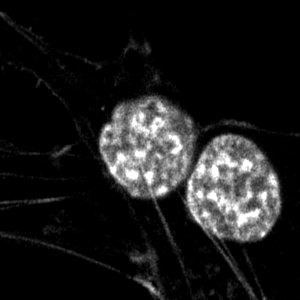

Supplement: Supplementary file 7 — Source data Fig. 5 [file 44321_2025_340_MOESM7_ESM.zip › Figure 5/Figure 5C/DNA Damage Images/PHENDC3 25/COMPOSITE DAPI ZOOM PHENDC3 25.jpg]

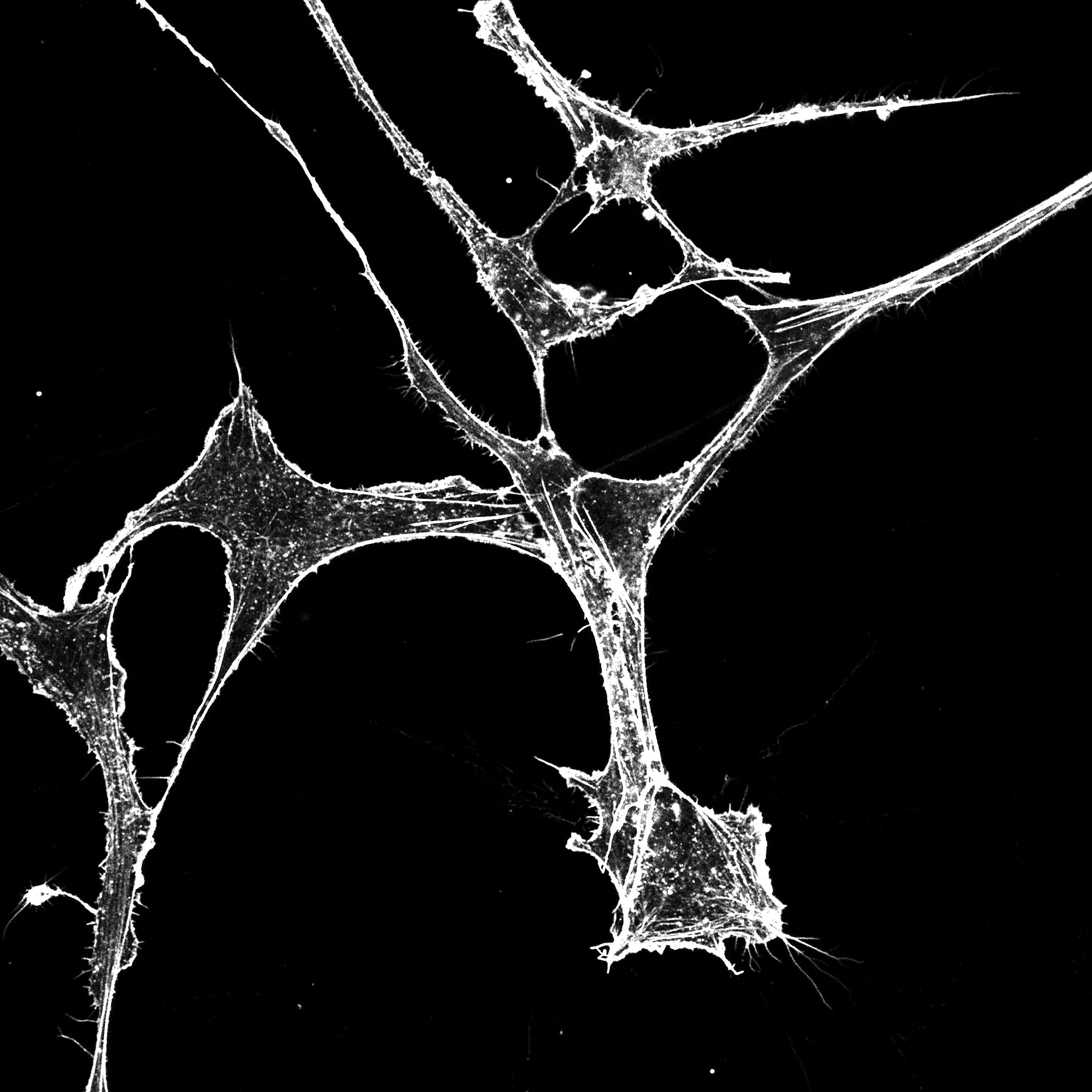

Supplement: Supplementary file 7 — Source data Fig. 5 [file 44321_2025_340_MOESM7_ESM.zip › Figure 5/Figure 5C/DNA Damage Images/PHENDC3 25/Composite F ACTIN PHENDC3 25.jpg]

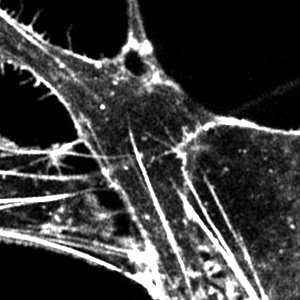

Supplement: Supplementary file 7 — Source data Fig. 5 [file 44321_2025_340_MOESM7_ESM.zip › Figure 5/Figure 5C/DNA Damage Images/PHENDC3 25/Composite F ACTIN ZOOM PHENDC3 25.jpg]

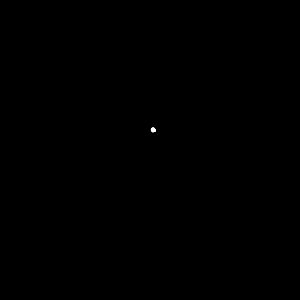

Supplement: Supplementary file 7 — Source data Fig. 5 [file 44321_2025_340_MOESM7_ESM.zip › Figure 5/Figure 5C/DNA Damage Images/PHENDC3 25/Composite H2AX PHENDC3 25 ZOOM.jpg]

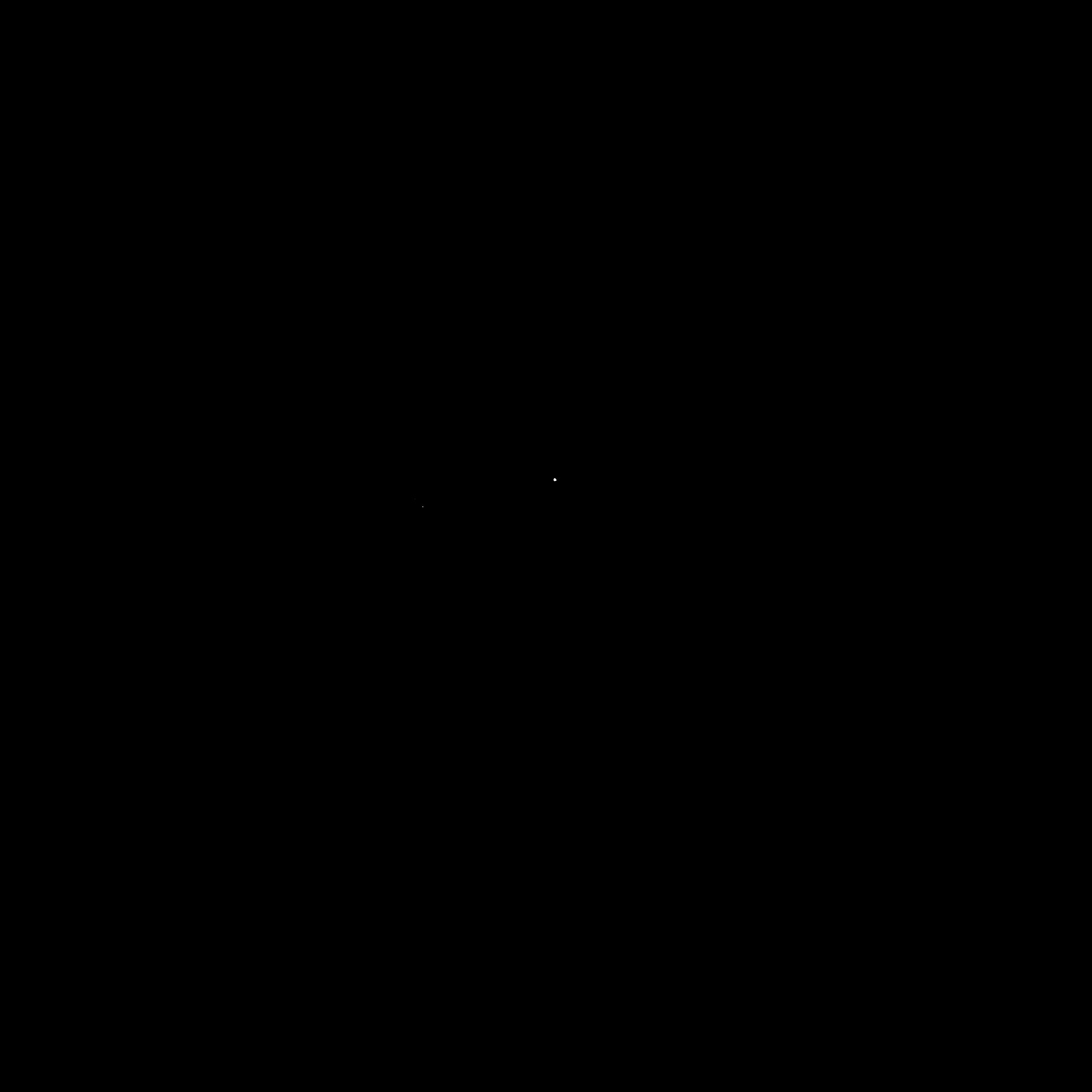

Supplement: Supplementary file 7 — Source data Fig. 5 [file 44321_2025_340_MOESM7_ESM.zip › Figure 5/Figure 5C/DNA Damage Images/PHENDC3 25/Composite H2AX PHENDC3 25.jpg]

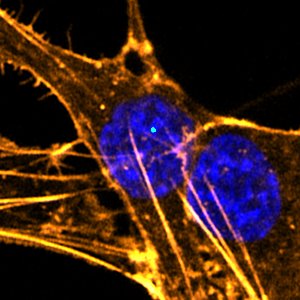

Supplement: Supplementary file 7 — Source data Fig. 5 [file 44321_2025_340_MOESM7_ESM.zip › Figure 5/Figure 5C/DNA Damage Images/PHENDC3 25/Composite MERGED PHENDC3 25 ZOOM.jpg]

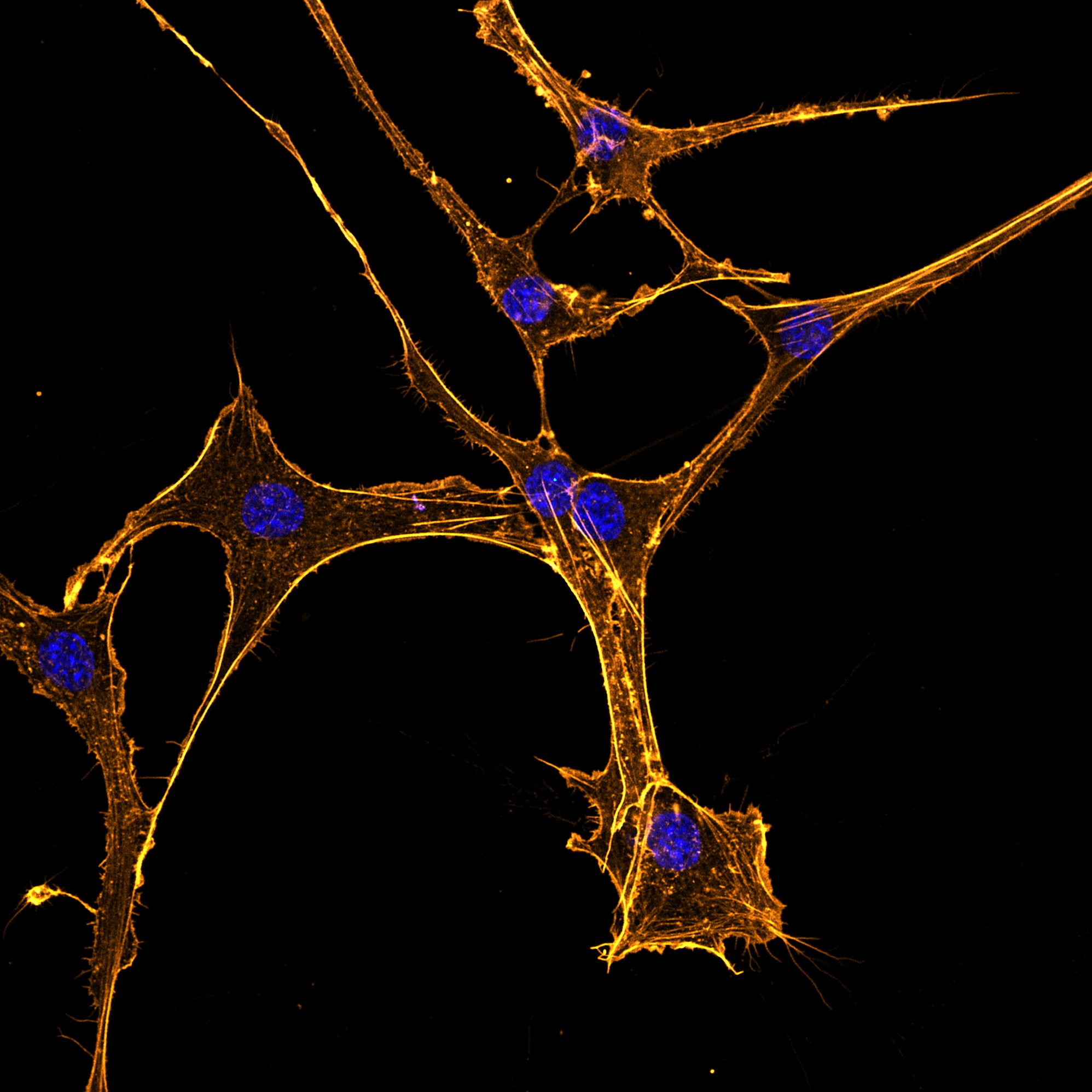

Supplement: Supplementary file 7 — Source data Fig. 5 [file 44321_2025_340_MOESM7_ESM.zip › Figure 5/Figure 5C/DNA Damage Images/PHENDC3 25/Composite MERGED PHENDC3 25.jpg]

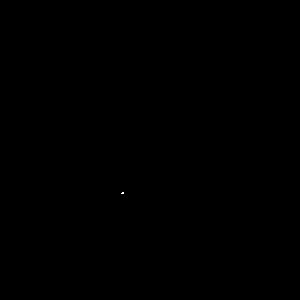

Supplement: Supplementary file 7 — Source data Fig. 5 [file 44321_2025_340_MOESM7_ESM.zip › Figure 5/Figure 5C/DNA Damage Images/PHENDC3 50/C2-Composite H2AX PHENDC3 50 ZOOM.jpg]

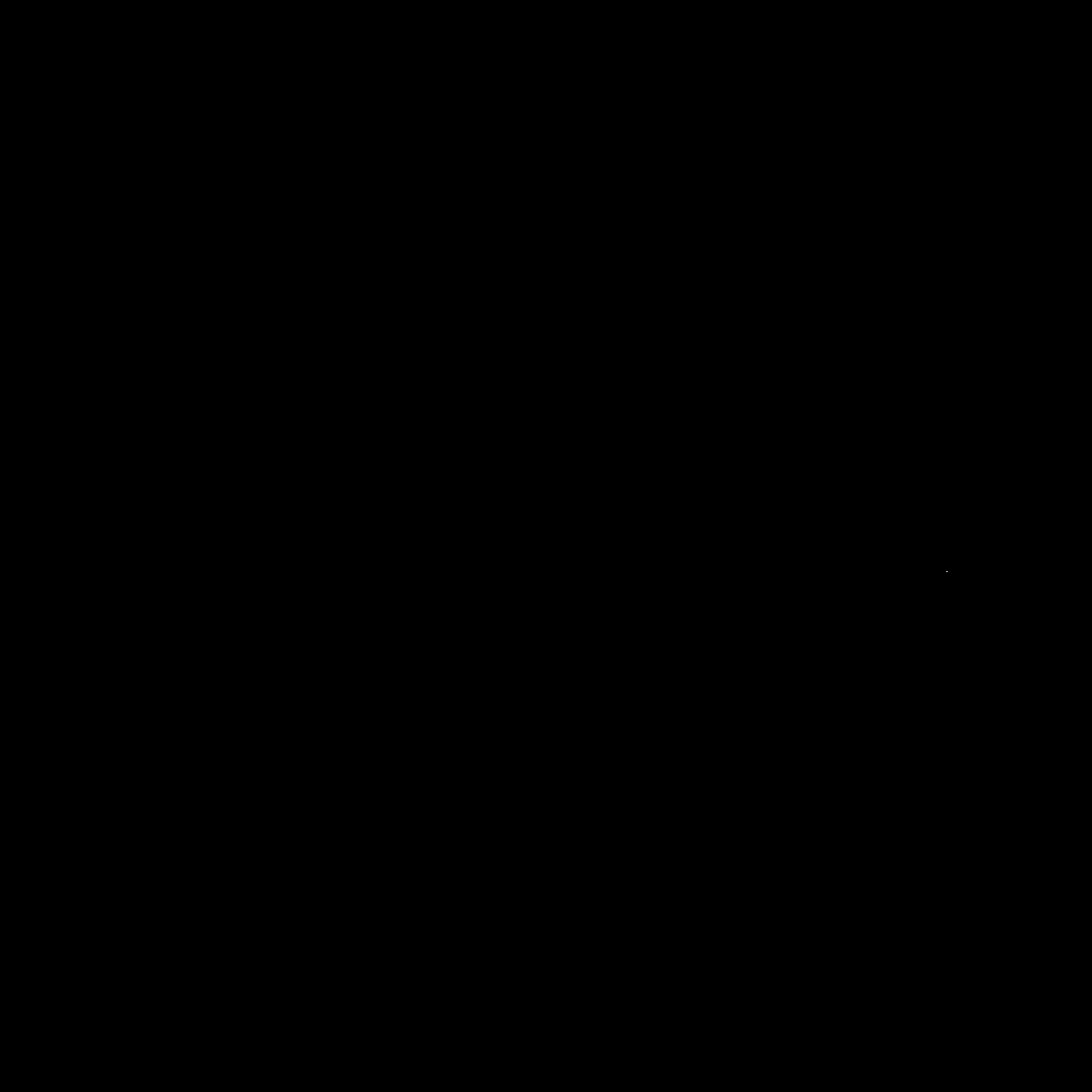

Supplement: Supplementary file 7 — Source data Fig. 5 [file 44321_2025_340_MOESM7_ESM.zip › Figure 5/Figure 5C/DNA Damage Images/PHENDC3 50/C2-Composite H2AX PHENDC3 50.jpg]

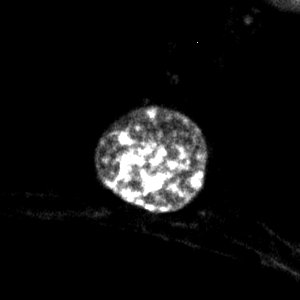

Supplement: Supplementary file 7 — Source data Fig. 5 [file 44321_2025_340_MOESM7_ESM.zip › Figure 5/Figure 5C/DNA Damage Images/PHENDC3 50/C3-Composite PHENDC3 50 DAPI ZOOM.jpg]

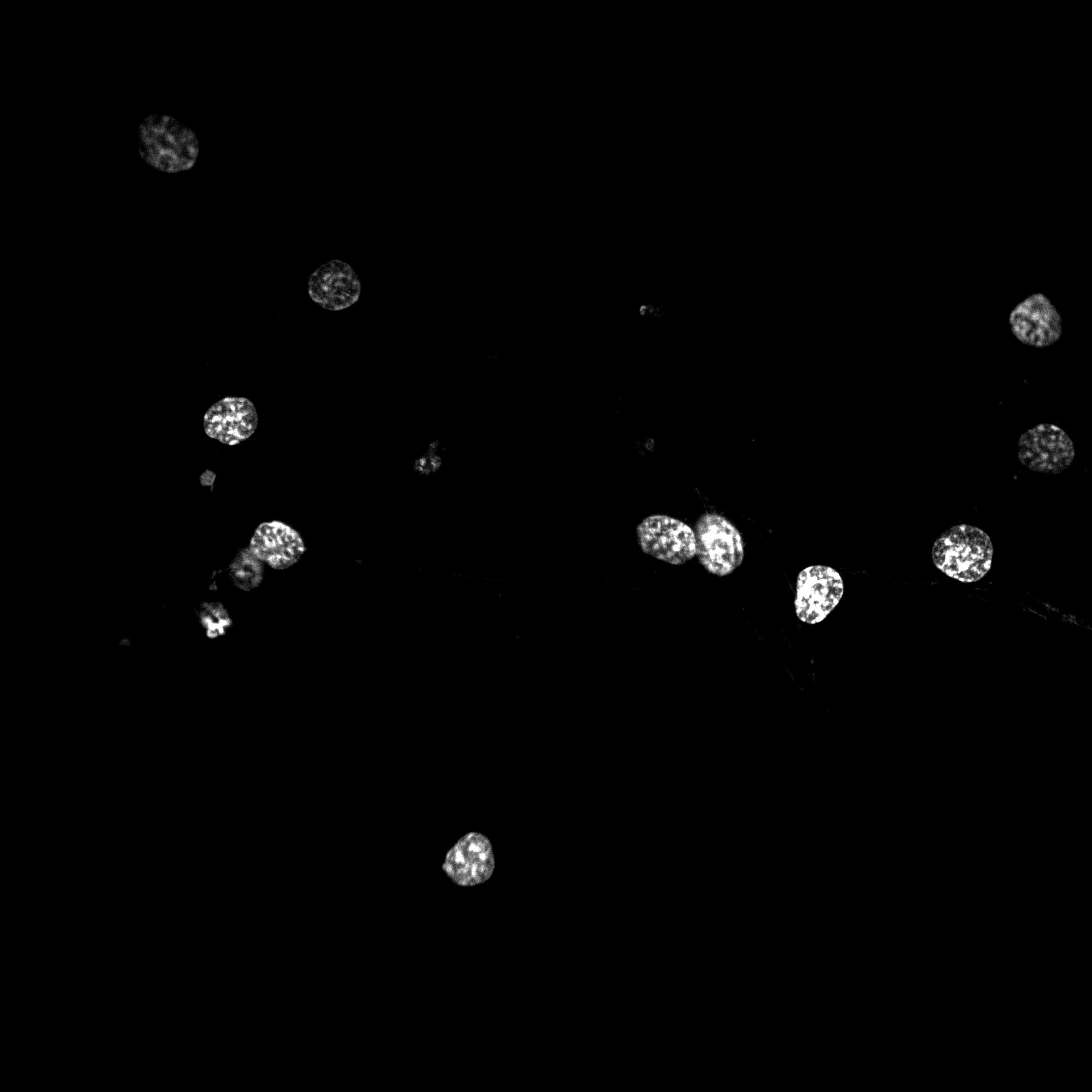

Supplement: Supplementary file 7 — Source data Fig. 5 [file 44321_2025_340_MOESM7_ESM.zip › Figure 5/Figure 5C/DNA Damage Images/PHENDC3 50/C3-Composite PHENDC3 50 DAPI.jpg]

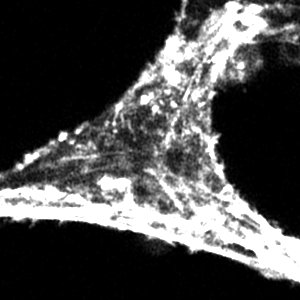

Supplement: Supplementary file 7 — Source data Fig. 5 [file 44321_2025_340_MOESM7_ESM.zip › Figure 5/Figure 5C/DNA Damage Images/PHENDC3 50/Composite F ACTIN PHENDC3 50 ZOOM.jpg]

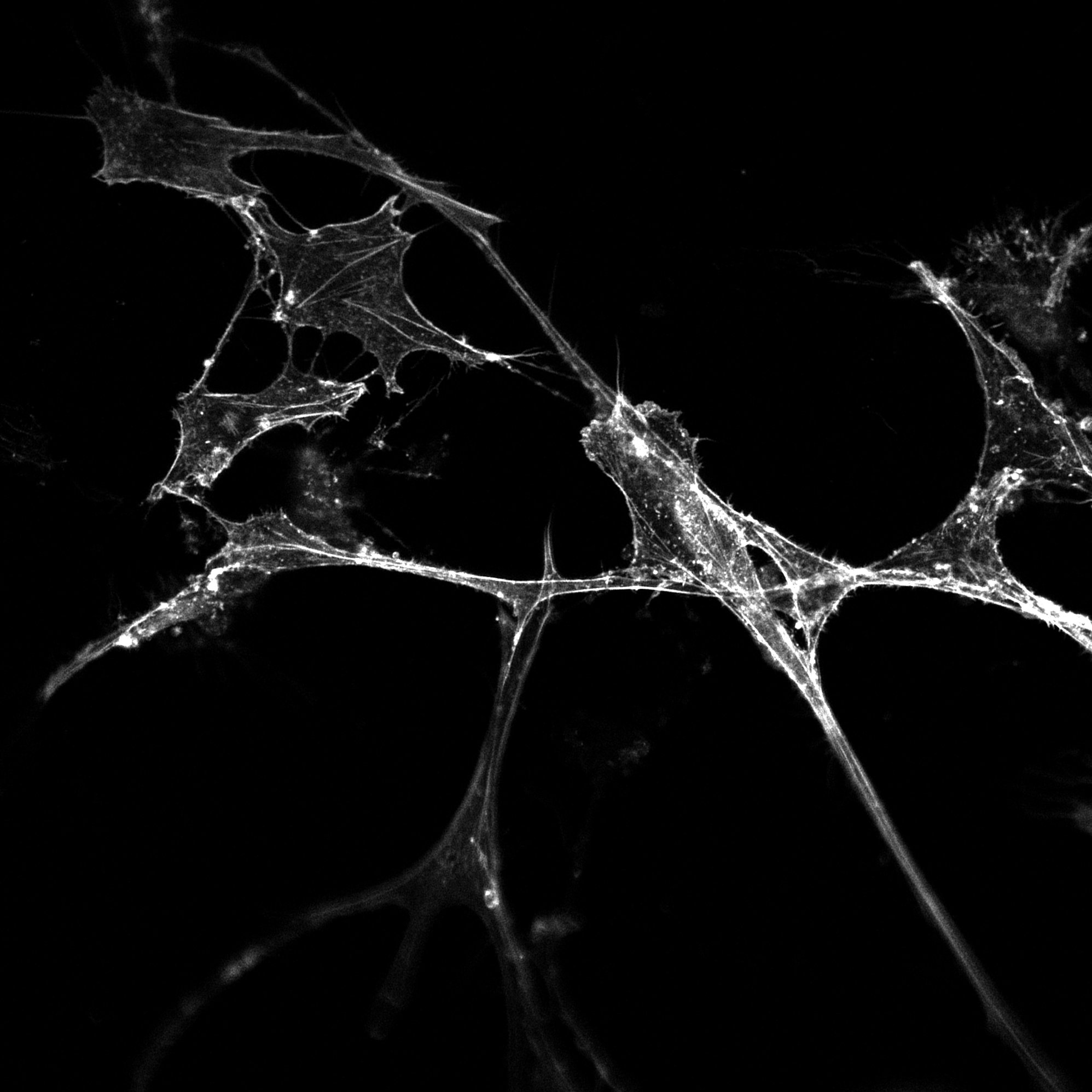

Supplement: Supplementary file 7 — Source data Fig. 5 [file 44321_2025_340_MOESM7_ESM.zip › Figure 5/Figure 5C/DNA Damage Images/PHENDC3 50/Composite F ACTIN PHENDC3 50.jpg]

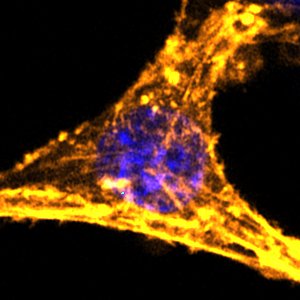

Supplement: Supplementary file 7 — Source data Fig. 5 [file 44321_2025_340_MOESM7_ESM.zip › Figure 5/Figure 5C/DNA Damage Images/PHENDC3 50/Composite MERGED PHENDC3 50 ZOOM.jpg]

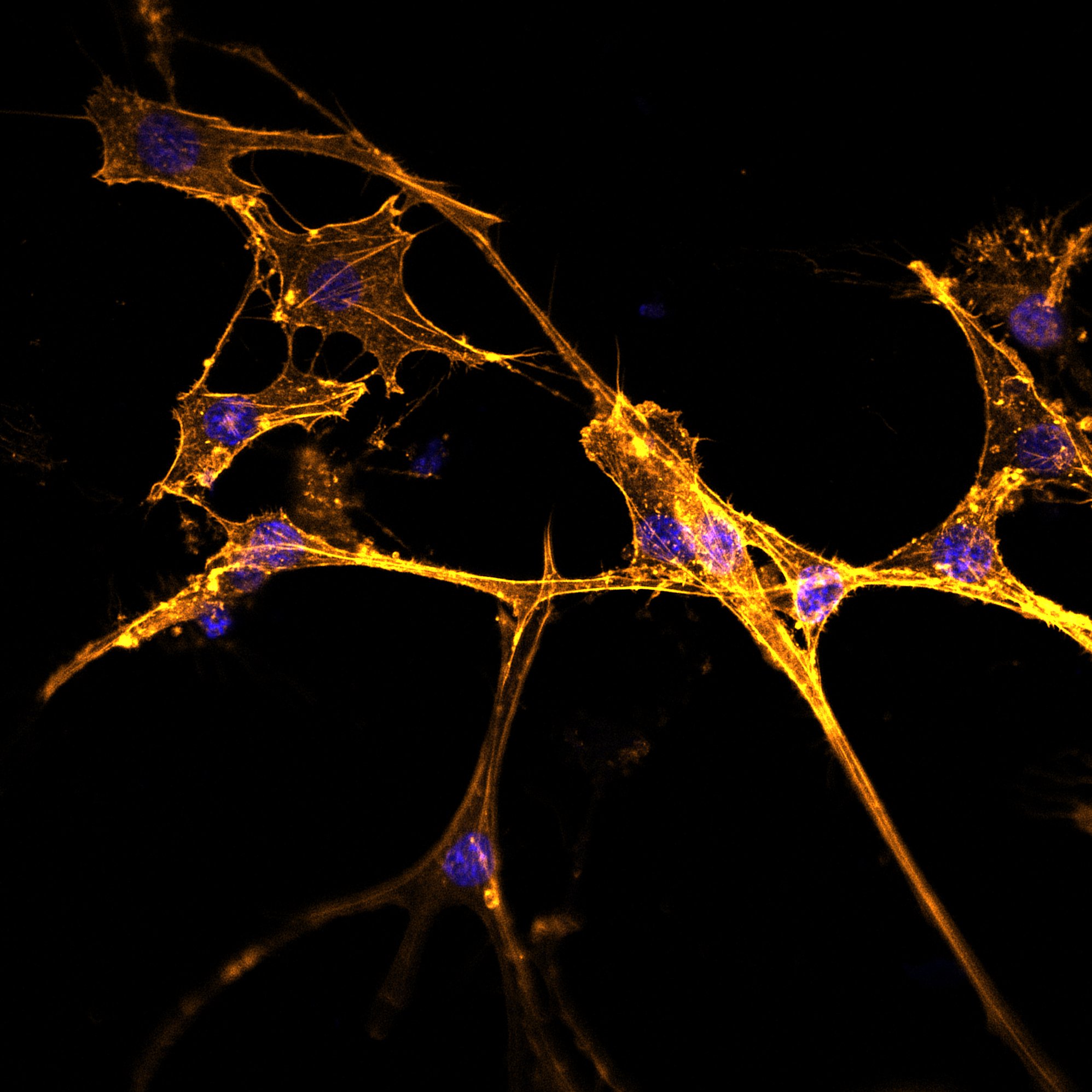

Supplement: Supplementary file 7 — Source data Fig. 5 [file 44321_2025_340_MOESM7_ESM.zip › Figure 5/Figure 5C/DNA Damage Images/PHENDC3 50/Composite MERGED PHENDC3 50.jpg]

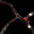

Supplement: Supplementary file 8 — Source data Fig. 6 [file 44321_2025_340_MOESM8_ESM.zip › Figure 6/Figure 6A/Merge PDS 1.56.tif]

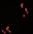

Supplement: Supplementary file 8 — Source data Fig. 6 [file 44321_2025_340_MOESM8_ESM.zip › Figure 6/Figure 6A/Merge PDS 12.5.tif]
