# Supplementary figures and images for: Evidence that G-quadruplexes form in pathogenic fungi and represent promising antifungal targets (part 2 of 2)
Source: EMBO Mol Med. 2025 Nov 17;17(12):3636–56. doi: 10.1038/s44321-025-00340-1 (PMC12686049; doi:10.1038/s44321-025-00340-1)

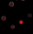

Supplement: Supplementary file 8 — Source data Fig. 6 [file 44321_2025_340_MOESM8_ESM.zip › Figure 6/Figure 6A/Merge PhenDC3 1.56.tif]

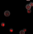

Supplement: Supplementary file 8 — Source data Fig. 6 [file 44321_2025_340_MOESM8_ESM.zip › Figure 6/Figure 6A/Merge PhenDC3 12.5.tif]

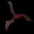

Supplement: Supplementary file 8 — Source data Fig. 6 [file 44321_2025_340_MOESM8_ESM.zip › Figure 6/Figure 6A/Merge untreated.tif]

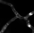

Supplement: Supplementary file 8 — Source data Fig. 6 [file 44321_2025_340_MOESM8_ESM.zip › Figure 6/Figure 6A/PDS 1.56 Hoechst.tif]

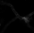

Supplement: Supplementary file 8 — Source data Fig. 6 [file 44321_2025_340_MOESM8_ESM.zip › Figure 6/Figure 6A/PDS 1.56 QUMA1.tif]

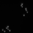

Supplement: Supplementary file 8 — Source data Fig. 6 [file 44321_2025_340_MOESM8_ESM.zip › Figure 6/Figure 6A/PDS 12.5 Hoechst.tif]

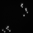

Supplement: Supplementary file 8 — Source data Fig. 6 [file 44321_2025_340_MOESM8_ESM.zip › Figure 6/Figure 6A/PDS 12.5 QUMA1.tif]

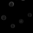

Supplement: Supplementary file 8 — Source data Fig. 6 [file 44321_2025_340_MOESM8_ESM.zip › Figure 6/Figure 6A/PhenDC3 1.56 Hoechst.tif]

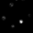

Supplement: Supplementary file 8 — Source data Fig. 6 [file 44321_2025_340_MOESM8_ESM.zip › Figure 6/Figure 6A/PhenDC3 1.56 QUMA1.tif]

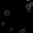

Supplement: Supplementary file 8 — Source data Fig. 6 [file 44321_2025_340_MOESM8_ESM.zip › Figure 6/Figure 6A/PhenDC3 12.5 Hoechst.tif]

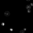

Supplement: Supplementary file 8 — Source data Fig. 6 [file 44321_2025_340_MOESM8_ESM.zip › Figure 6/Figure 6A/PhenDC3 12.5 QUMA1.tif]

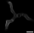

Supplement: Supplementary file 8 — Source data Fig. 6 [file 44321_2025_340_MOESM8_ESM.zip › Figure 6/Figure 6A/Untreated Hoechst.tif]
